# Supplementary material for: Exploring women’s development group leaders’ support to maternal, neonatal and child health care: A qualitative study in Tigray region, Ethiopia
Source: PLoS One. 2021 Sep 23;16(9):e0257602. doi: 10.1371/journal.pone.0257602 (PMC8460027; doi:10.1371/journal.pone.0257602)
Supplement: S1 File — (PDF) [file pone.0257602.s001.pdf]

**Research title:** Exploring women's development group leaders' support to maternal, neonatal and child health care: a qualitative study in Tigray region, Ethiopia, 2018

Guidance's prepared based on thematic

## Focus Group Discussion (FGD) Guide: Women Development Group (WDG) Leaders

### Introduction

Thank you for completing the informed consent form and for taking the time to speak with me today. I have several questions to ask you that I have prepared in advance. I will turn on the tape recorder now. If you have any additional questions or comments as we do the interview, please feel free to share them with me.

### Theme 1 Section One: FGD Details

### FGD information

1.1 FGD ID: ___________

1.2 FGD date: ___________

1.3 FGD start time: ___________

1.4 FGD end time: ___________

1.5 Interviewer code: ___________

1.6 Note taker code: ___________

1.7 Translator code: ___________

1.8 Tape recording number: _______

Theme 1: Socio-demographic

| Respondent number | Age | Marital status | Educational status | Parity | Position (1to30 or 1to 5) | Working in position in months |
|---|---|---|---|---|---|---|
| | | | | | | |
| | | | | | | |
| | | | | | | |
| | | | | | | |
| | | | | | | |
| | | | | | | |
| | | | | | | |
| | | | | | | |

Section C**: Facilitators and barriers of the WDG leaders of performance**

| Main questions | Probing questions (Facilitators and barriers) |
|---|---|
| **Theme 2**: **Understanding and perception of WDG role in MNCH care** | |
| 2.1 What is your responsibility in the community you are living in? | Health, Agriculture & water, education Security& governance etc. |
| 2.2 Could you tell me about your main activities as a WDG leader in MNCH? | 1. Way of Planning, meeting and referral/ reporting with HEWs: (Phone, questioning and answering, participatory at all levels, Timing, down –up, support by paper or pictorial format.<br>2. Organize pregnant women conference: - Who is leading PWC?<br>3. Discuss about referrals for pregnant women:<br>4. Discuss about referrals for recently delivered mothers and their newborns: On what they discussed?<br>5. Provide House visit:<br>6. Conduct health related campaigns: |
| **Notes:** to understand the WDG leaders' role and perception of WDG leaders in their work and performance. | |
| **Thematic 3. Knowledge of WDG leaders on MNCH care** | |
| 3.1 Do you think that you have adequate knowledge to provide promotive and preventive MNCH care as WDG leaders?<br><br>3.2 What knowledge gaps might be limiting your responsibility as a WDG leader? | Could you elaborate/ mention me the following health domains: Probe? |
| **Thematic 4. Training of WDG leaders on MNCH care** | |
| 4.1 How do you see the training opportunities?<br>4.2 If not received the training in the last year, could you tell me the reason/s?<br>4.3 How you receive the basic training from HEWs? | Could you tell me, how was conducted the ways of the training Probe? |
| **Thematic 5. Supervision of WDG leaders on MNCH care** | |
| 5.1 How do you see the supervision carried out? | • Probe? |

| | |
|---|---|
| 5.2 Could you tell me, the opinion of the supervision provided by HEWs? | |
| **Thematic 6. Recognition of WDG leaders** | |
| 6.1 How do you see the recognition or appreciation mechanisms made for your work as WDG leader from different health system and community levels? | (Based on: Principles, Participatory, Transparency and others)<br>• Probe? |
| **Thematic 7. Environment work- structure and hierarchy** | |
| 7.1 What is your opinion of WDG structure including hierarchy?<br>**7.2** Network WDG leaders with HEWs or other actors recruited, | • Probe? |
| **Thematic 8. Trust of WDG members and other stakeholders for WDG leaders** | |
| 8.1 Could you tell me have the community trusts the WDG leaders? | Could you tell me your own experience?<br>Probe? |
| **Thematic 9. Linkage WDG leaders with other stakeholders** | |
| 9.1 Could you tell me your linkages and expectations from the different stakeholders on MNCH care? | • Probe? |
| How do you see your work as a WDG leader? | • Your performance in the job<br>• Satisfaction with your performance<br>• Things to be improved |

**Notes:** We want to describe and identify facilitators and barriers of practice in related with linkage of HEWs, command post and traditional birth attendants as well as traditional healers. And we want to describe and identify facilitators and barriers mainly under knowledge, training, supervision, recognition and environmental work. Also, we want to compare the actual manual demanded and what they are exercising and their expectation.

**End of module.**

**Thank you.**

**Research title:** Exploring women's development group leaders' support to maternal, neonatal and child health care: a qualitative study in Tigray region, Ethiopia, 2018

## Focus Group Discussion (FGD) Guide: HEWs

### Introduction

Thank you for completing the informed consent form and for taking the time to speak with me today. I have several questions to ask you that I have prepared in advance. I will turn on the tape recorder now. If you have any additional questions or comments as we do the interview, please feel free to share them with me.

### Theme 1 Section One: FGD information

1.1 FGD ID: ___________

1.2 FGD date: ___________

1.3 FGD start time: ___________

1.4 FGD end time: ___________

1.5 Interviewer code: ___________

1.6 Note taker code: ___________

1.7 Translator code: ___________

1.8 Tape recording number: _______

### Theme 1: Socio-demographic

| Respondent number | Educational status | Profession | Years of working | Remark |
|---|---|---|---|---|
| | | | | |
| | | | | |
| | | | | |
| | | | | |
| | | | | |
| | | | | |
| | | | | |
| | | | | |

Section C**: Facilitators and barriers of the WDG leaders of performance**

| Main questions | Probing questions |
|---|---|
| **Theme 2**: **Understanding and perception of WDG role in MNCH care** | |
| 2.1 What is the responsibility of WDG leaders in the community they are living in? | Health, Agriculture & water, education Security& governance etc. |
| 2.2 Could you tell me about the main activities of WDG leaders in MNCH? | Probe?<br>1. Way of Planning, Meeting and referral/ reporting with HEWs: Questioning and answering, Timing, participatory at all levels, down –up, support by paper or pictorial format.<br>2. Organize pregnant women conference: - Who is leading PWC?<br>3. Discuss about referrals for pregnant women:<br>4. Discuss about referrals for recently delivered mothers and their newborns: On what they discussed?<br>5. Provide House visit:<br>6. Conduct health related campaigns: the 1 to 5 network WDG leaders, 1 to 30 WDG leaders, for example, Polio, trachoma and others. |
| **Notes:** to understand the WDG leaders' role and perception of WDG leaders in their work and performance. | |
| **Thematic 3. Knowledge of WDG leaders on MNCH care** | |
| 3.1 Do you think the WDG leaders have adequate knowledge to provide promotive and preventive MNCH care?<br>3.2 What knowledge gaps might be limiting their responsibility as a WDG leader? | Could you elaborate/ mention me the following health domains:<br>Probe? |
| **Thematic 4. Training of WDG leaders on MNCH care** | |

| | |
|---|---|
| 4.1 How do you see the training opportunities?<br>4.2 If the WDG leaders not received the training in the last year, could you tell me the reason/s?<br>4.3 How you give the basic training for WDG leaders? | Could you tell me, how was conducted the ways of the training? Probe? |
| **Thematic 5. Supervision of WDG leaders on MNCH care** | |
| 5.1 How do you see the supervision carried out to WDG leaders?<br>5.2 Could you tell me, your opinion on how you are providing the supervision? | • Probe? |
| **Thematic 6. Recognition of WDG leaders** | |
| 6.1 How do you see the recognition or appreciation mechanisms made for WDG leader for their work from different health system and community levels? | (Based on: Principles, Participatory, Transparency) Probe?<br>• **Health system**:<br>• **Community system**: |
| **Thematic 7. Environment work- structure and hierarchy** | |
| 7.3 What is your opinion in WDG structure including hierarchy?<br>7.2 What is your opinion on recruited of network and WDG leaders , | |
| **Thematic 8. Trust of WDG members and other stakeholders for WDG leaders** | |
| 8.1 Could you tell me, have the community trusts the WDG leaders?<br>8.2 How do you see the relationship and communication among WDG members, network leaders and 1 to 30 WDG leaders? | Could you tell me your own experience?<br>Source of information,<br>Relationship<br>Communication |
| **Thematic 9. Linkage WDG leaders with other stakeholders** | |

| | |
|---|---|
| 9.1 Could you tell me the WDG leaders' linkage from the view of different stakeholders on MNCH care? | Probe?<br>• Community level<br>• Government level (Health system: |
| How do you see their work as a WDG leader? | • Their performance in the job<br>• Satisfaction with the compare the performance of WDG<br>• Things to be improved |

**Notes:** We want to describe and identify facilitators and barriers of practice in related with linkage of HEWs, command post and traditional birth attendants as well as traditional healers. And we want to describe and identify facilitators and barriers mainly under knowledge, training, supervision, recognition and environmental work. Also, we want to compare the actual manual demanded and what they are exercising and their expectation.

**End of module.**

**Thank you.**

**Research title:** Exploring women's development group leaders' support to maternal, neonatal and child health care: a qualitative study in Tigray region, Ethiopia, 2018

## <u>In-Depth-Interview Guide: WDG leaders</u>

### Introduction

Thank you for completing the informed consent form and for taking the time to speak with me today. I have several questions to ask you that I have prepared in advance. I will turn on the tape recorder now. If you have any additional questions or comments as we do the interview, please feel free to share them with me.

## Theme 1 Section One: Interview Details

### Section A: Interview details

1. Region:_________________________
2. Zone: _________________________
3. Woreda: _________________________
4. Tabia: _________________________
5. Name of IDI interviewee : _________________________
6. Institution of interviewee: __________________________
7. Interviewer name: _________________________
8. Interview Date _________________________
9. Interview start time: _________________________
10. Interview end time: _________________________

### Section B: Interviewee Professional Information

1. ID. ____________________________
2. Place of residence (Rural/ urban): __________
3. Marital status: __________
4. Sex: __________
5. Religion: __________
6. Education: __________
7. participant's age in year: __________
8. How many women serving: __________
9. Experience in year: _________
10. Interviewer code: _________________________
11. Tape Recording number:__________

Section C**: Facilitators and barriers of the WDG leaders of performance**

| Main questions | Probing questions (Facilitators and barriers) |
|---|---|
| **Theme 2**: **Understanding and perception of WDG role in MNCH care** | |
| 2.1 What is your responsibility in the community you are living in? | Others…(specify) |
| 2.2 Could you tell me about your main activities as a WDG leader in health?<br>2.3 Could you tell me you're the activities and carried out as a WDG leader in MNCH care, please? | 1. Way of Planning, meeting and referral/ reporting with HEWs: (Phone, questioning and answering, participatory at all levels, Timing, down –up, support by paper or pictorial format.<br>At what interval, you meet:<br>• With their all members of the network (6)_____<br>• With their all members of the WDG (30)______<br>• Network leaders themselves as a WDG (5)_____ and<br>• One to five leaders With HEWs____<br>• One to 30 leaders with HEWs_____<br>2. Organize pregnant women conference: - Who is leading PWC?<br>- How are you doing immediate identification of PW?<br>- Do you think all expected pregnant women attend in PWC? If not why?<br>- What agendas set and discuss?<br>3. Discuss about referrals for pregnant women:<br>- Do you do a discussion with PW?<br>If yes, in what main topics:<br>-Importance of early ANC and FANC,<br>-HIV test and improve feeding practice,<br>-Readiness: save money, clothes for both, conscious traditional ambulance and PW save telephones of network WDG leaders. HEW and other Health task forces.<br>- Danger signs of PW? (Vaginal bleeding, headache, dizziness, Preeclampsia)<br>- Waiting room<br>4. Discuss about referrals for recently delivered mothers and their newborns: On what they discussed?<br>-delivered mother and newborn care : PNC follow up,<br>- Newborn danger signs, and<br>- Child danger signs<br>5. Provide House visit:<br>- Assess identification and immediate tracing PW, |

| | |
|---|---|
| | - mother and newborn care follow up and child care including vaccination, Growth monitoring, sanitation and hygiene etc,<br>6. Conduct health related campaigns: the 1 to 5 network WDG leaders, 1 to 30 WDG leaders, for example, Polio, trachoma and others. |
| **Notes:** to understand the WDG leaders' role and perception of WDG leaders in their work and performance. | |
| **Thematic 3. Knowledge of WDG leaders on MNCH care** | |
| 3.1 Do you think that you have knowledge to provide promotive and preventive **health** care as WDG leaders?<br>3.2 Do you think that you have adequate knowledge to provide promotive and preventive **MNCH** care as WDG leaders?<br>3.3 Do you think that you have knowledge to provide promotive and preventive **newborn** care as WDG leaders?<br><br>3.4 What knowledge gaps might be limiting your responsibility as a WDG leader? | Could you elaborate/ mention me the following health domains:<br>-Danger signs of PW?<br>-PNC follow up: When and what types of care you know for both delivered mothers and newborns?<br>- Newborn care and danger signs, and<br>- Child danger signs |
| **Thematic 4. Training of WDG leaders on MNCH care** | |
| 4.1 How do you see the training opportunities?<br>4.2 If not received the training in the last year, could you tell me the reason?<br>4.3 How you receive the basic training from HEWs? | Could you tell me, how was conducted the ways of the training<br>• How frequent did you get training in a year?<br>• How regular is/are the training/s you get?<br>• For how long day/s or hours you received the training?<br>• Have HEWs received TOT, availability of WDG manual/ protocol training, conducive environment for training,<br>- Did you receive the training with respect to maternal, newborn and child care?<br>- What sort of trainings did you get?<br>- How you receive training on how to sort the activities to HEWs<br>- How you explain whether or not you were satisfied with the MNCH care training received? |
| **Thematic 5. Supervision of WDG leaders on MNCH care** | |
| 5.1 How do you see the supervision carried out? | • Have you seen supervision protocol or guideline for WDG leaders on MNCH,<br>• shortage of supervision materials<br>• How you think the HEWs or HWs received skill of supervision before give a training? |

| | |
|---|---|
| 5.2 Could you tell me, the opinion of the supervision provided by HEWs? | • Are you convinced the HEWs have adequate knowledge to conduct the supervision?<br>• How frequent do HEWs make supervision?<br>• Do they use checklist?<br>• For whom do you give feedback? WDG leaders, administration, Women affaire, coalition and others |
| **Thematic 6. Recognition of WDG leaders** | |
| 6.1 How do you see the recognition or appreciation mechanisms made for your work as WDG leader from different **health** and **community system** levels? (Based on following: fairness Principles, Participatory, Transparency and others)<br>6.2 Could you tell have the recognition given for the general health performance?<br>6.3 How explain the recognition in MNCH care? | (Based on: Fairness, Principles, Participatory, Transparency and others)<br>• **Health system**: HEWs, Health center, Woreda, regional, partners (specify, FMoH)<br>• **Community system**: Women steering committee, coalition, Administration/ command post, individual member of community, Edir or other stakeholders |
| **Thematic 7. Environment work- structure and hierarchy** | |
| 7.4 What is your opinion of WDG structure including hierarchy?<br>7.2 Network WDG leaders with HEWs or other actors recruited, | • How you evaluate the proportion network WDG leaders with HEWs recruited? If yes,<br>• How affect the disproportion of network WDG leaders with HEWs to supervision, training. Recognition and others?<br>• Could you tell me the structural hierarchy that practiced?<br>Could you tell me ways of nomination mechanism for WDG leaders?<br>• Model family graduated<br>• Who is/ are active participant/s while nomination WDG leaders?<br>• Freedom to nominate<br>• Somebody submit proposal to be acted as a WDG leader<br>• Somebody who are considered as charge to be decided the official nominated by themselves without knowledge of the beneficiaries,<br>Could you tell me ways of firing of mechanism for WDG leaders?<br>• Who is/ are active participant/s while fire WDG leaders?<br>• Freedom (participatory)<br>• Somebody submit proposal for the WDG leader to be fired. |

| | |
|---|---|
| | • Somebody who are considered as charge to be fired themselves without knowledge of the members, |
| **Thematic 8. Trust of WDG members and other stakeholders for WDG leaders** | |
| 8.1 Could you tell me have the community trusts the WDG leaders? | Could you tell me your own experience?<br>Source of information,<br>What about among WDG members, network leaders and 1 to 30 WDG leaders? |
| **Thematic 9. Linkage WDG leaders with other stakeholders** | |
| 9.1 Who is/ are working with you?<br>9.2 If you could mention, in which activities support you?<br>9.3 Could you tell me your linkages and expectations from the different stakeholders on MNCH care? | • Community level (Women steering committee, women association, Women coalition, Male development group, youth association, farmer association, Social organizations, like, Edir, Traditional healers, Culture of the community, Religion and religious leaders, Influential people, TBA and others),<br>• Government level (Health system: HEWs, HEW supervisors, HC and woreda health office, Administration, women affaire, Education, agriculture, water and others). What do you think to strengthen WDG? Motivation: Incentives (In kind or/and financial), recognition, training, fellow up, supervision, disseminations health information to their followers and others. |
| How do you see your work as a WDG leader? | • Your performance in the job<br>• Satisfaction with your performance<br>• Things to be improved |

**Notes:** We want to describe and identify facilitators and barriers of practice in related with linkage of HEWs, command post and traditional birth attendants as well as traditional healers. And we want to describe and identify facilitators and barriers mainly under knowledge, training, supervision, recognition and environmental work. Also, we want to compare the actual manual demanded and what they are exercising and their expectation.

**End of module.**

**Thank you.**

**Research title:** Exploring women's development group leaders' support to maternal, neonatal and child health care: a qualitative study in Tigray region, Ethiopia, 2018

**Key-Informative-Interview Guide: HEW supervisors, Woreda experts (HEP and MNCH) and Regional (HEP coordinator and MNCH coordinator)**

**Introduction**

Thank you for completing the informed consent form and for taking the time to speak with me today. I have several questions to ask you that I have prepared in advance. I will turn on the tape recorder now. If you have any additional questions or comments as we do the interview, please feel free to share them with me.

**Theme 1 Section One: Interview Details**

**Section A: Interview details**

1. Region:_________________________
2. Zone: _________________________
3. Woreda: _________________________
4. Tabia: _________________________
5. Name of KII interviewee : _________________________
6. Institution of interviewee: __________________________
7. Interviewer name: _________________________
8. Interview Date _________________________
9. Interview start time: _________________________
10. Interview end time: _________________________

**Section B: Interviewee Professional Information**

1. ID. ____________________________
2. Place of residence (Rural/ urban): __________
3. Marital status: __________
4. Sex: _________
5. Religion: __________
6. Profession: _________
7. Experience in year: _________
8. Interviewer code: _________________________
9. Tape Recording number:__________

Section C**: Facilitators and barriers of the WDG leaders of performance**

| Main questions | Probing questions |
|---|---|
| **Theme 2**: **Understanding and perception of WDG role in MNCH care** | |
| 2.1 What is the responsibility of WDG leaders in the community they are living in? | Health, Agriculture & water, education Security& governance etc. |
| 2.2 Could you tell me about the main activities of WDG leaders in MNCH? | 1. Way of Planning, Meeting and referral/ reporting with HEWs: Questioning and answering, Timing, participatory at all levels, down –up, support by paper or pictorial format.<br>At what interval, they meet:<br>• With their all members of the network (6)_____<br>• With their all members of the WDG (30)______<br>• Network leaders themselves as a WDG (5)_____ and<br>• One to five leaders With HEWs____<br>• One to 30 leaders with HEWs_____<br>2. Organize pregnant women conference: - Who is leading PWC?<br>- How are you doing immediate identification of PW?<br>- Did you think perform monthly with expected pregnant women? If not why?<br>- What agendas set and discuss?<br>3. Discuss about referrals for pregnant women:<br>- Did you do a discussion with PW?<br>If yes, in what main topics:<br>-Importance of early ANC and FANC,<br>-HIV test and improve feeding practice,<br>-Readiness: save money, clothes for both, conscious traditional ambulance and PW save telephones of network WDG leaders. HEW and other Health task forces. |

| | |
|---|---|
| | -Awareness on danger signs of PW?<br>4. Discuss about referrals for recently delivered mothers and their newborns: On what they discussed?<br>-delivered mother: PNC follow up,<br>- Newborn care and danger signs, and<br>- Child danger signs<br>5. Provide House visit:<br>- Assess identification and immediate tracing PW,<br>- mother newborn care follow up and child care including vaccination, Growth monitoring, sanitation and hygiene etc,<br>6. Conduct health related campaigns: the 1 to 5 network WDG leaders, 1 to 30 WDG leaders, for example, Polio, trachoma and others. |
| **Notes:** to understand the WDG leaders' role and perception of WDG leaders in their work and performance. | |
| **Thematic 3. Knowledge of WDG leaders on MNCH care** | |
| 3.1 Do you think the WDG leaders have adequate knowledge to provide promotive and preventive MNCH care?<br><br>3.2 What knowledge gaps might be limiting their responsibility as a WDG leader? | Could you elaborate/ mention me the following health domains:<br>-Danger signs of PW?<br>-PNC follow up: When and what types of care you know for both delivered mothers and newborns?<br>- Newborn care and danger signs, and<br>- Child danger signs |
| **Thematic 4. Training of WDG leaders on MNCH care** | |

| | |
|---|---|
| 4.1 How do you see the training opportunities?<br>4.2 If the WDG leaders not received the training in the last year, could you tell me the reason?<br>4.3 How they receive the basic training from HEWs? | Could you tell me, how was conducted the ways of the training<br>• How frequent did they get training in a year?<br>• How regular is/are the training/s they get?<br>• For how long day/s or hours they received the training?<br>• Have HEWs received TOT, availability of WDG manual/ protocol training, conducive environment for training,<br>- Did they receive the training with respect to maternal, newborn and child care?<br>- What sort of trainings did you get?<br>- How did they receive training on how to sort the activities to HEWs<br>- How you explain whether or not they were satisfied with the MNCH care training received? |
| **Thematic 5. Supervision of WDG leaders on MNCH care** | |
| 5.1 How do you see the supervision carried out?<br><br>5.2 Could you tell me, your opinion on how the HEWs are providing the supervision? | • Have you seen supervision protocol or guideline for WDG leaders on MNCH,<br>• shortage of supervision materials<br>• How you think the HEWs or HWs received skill of supervision before give a training?<br>• Are you convinced the HEWs have adequate knowledge to conduct the supervision?<br>• How frequent do HEWs make supervision?<br>• Do they use checklist?<br>• For whom do you give feedback? WDG leaders, administration, Women affaire and others |
| **Thematic 6. Recognition of WDG leaders** | |

| | |
|---|---|
| 6.1 How do you see the recognition or appreciation mechanisms made for WDG leader for their work from different health system and community levels? | (Based on: Principles, Participatory, Transparency)<br>• **Health system**: HEWs, Health center, Woreda, regional, partners (specify)<br>• **Community system**: Women steering committee and Women coalition, Administration/ command post, individual member of community, Edir or others. |
| **Thematic 7. Environment work- structure and hierarchy** | |
| 7.5 What is your opinion in WDG structure including hierarchy?<br>7.2 What is your opinion on recruited of network and WDG leaders , | • How you evaluate the proportion network WDG leaders with HEWs recruited?<br>• How affect the disproportion of network WDG leaders with HEWs recruited to supervision, training. Recognition and others?<br>• Could you tell me the structural hierarchy that practicing?<br>Could you tell me ways of nomination mechanism for WDG leaders?<br>• Model family graduated<br>• Who is/ are active participant/s while nomination WDG leaders?<br>• Freedom to nominate<br>• Somebody submit proposal to be acted as a WDG leader<br>• Somebody who are considered as charge to be decided the official nominated by themselves without knowledge of the beneficiaries,<br>Could you tell me ways of fire mechanism for WDG leaders?<br>• Who is/ are active participant/s while fire WDG leaders?<br>• Freedom to fire network and WDG leaders<br>• Somebody submit proposal to be acted as a WDG leader<br>• Somebody who are considered as charge to be fired themselves without knowledge of the members, |
| **Thematic 8. Trust of WDG members and other stakeholders for WDG leaders** | |

| | |
|---|---|
| 8.1 Could you tell me, have the community trusts the WDG leaders?<br>8.2 How do you see the relationship and communication among WDG members, network leaders and 1 to 30 WDG leaders? | Could you tell me your own experience?<br>Source of information,<br>Relationship<br>Communication |
| **Thematic 9. Linkage WDG leaders with other stakeholders** | |
| 9.1 Could you tell me the WDG leaders' linkage from the view of different stakeholders on MNCH care? | • Community level (Women steering committee, women association, Women coalition, Male development group, youth association, farmer association, Social organizations, like, Edir, Traditional healers, Culture of the community, Religion and religious leaders, Influential people, TBA and others),<br>• Government level (Health system: HEWs, HEW supervisors, HC and woreda health office, Administration, women affaire, Education, agriculture, water and others). What do you think to strengthen WDG? Motivation: Incentives (In kind or/and financial), recognition, training, fellow up, supervision, disseminations health information to their followers and others. |
| How do you see their work as a WDG leader? | • Their performance in the job<br>• Satisfaction with the  compare the performance of WDG<br>• Things to be improved |

**Notes:** We want to describe and identify facilitators and barriers of practice in related with linkage of HEWs, command post and traditional birth attendants as well as traditional healers. And we want to describe and identify facilitators and barriers mainly under knowledge, training, supervision, recognition and environmental work. Also, we want to compare the actual manual demanded and what they are exercising and their expectation.

**End of module.**

**Thank you.**
