# Supplementary material for: Exploring women’s development group leaders’ support to maternal, neonatal and child health care: A qualitative study in Tigray region, Ethiopia
Source: PLoS One. 2021 Sep 23;16(9):e0257602. doi: 10.1371/journal.pone.0257602 (PMC8460027; doi:10.1371/journal.pone.0257602)
Supplement: S2 File — (PDF) [file pone.0257602.s002.pdf]

ርእሲ መፅናዒቲዕ፡ መሳለጥትን መዐንቀፍትን ንኣመራርሓ ልምዓት ጉጅለ ደቂ ኣንስትዮ ኣብ ምምሕያሽ ኣፈፃፅማ ክንክን ኣዴታት፣ ሓናጡን ህፃናትን፤ ትግራይ፤ ኢትዮጵያ 2010ዓ.ም

<u>ትኩር ጉጅለ ምይይጥ /ትጉም/፡ መፅናዒቲ ኣብ ኣመራርሓ ልምዓት ጉጅለ ደቂ ኣንስትዮ</u>

መእተዊ

ንቲ ወረቐት ወለንታ ኣሚንክናሉ ምምላእኺን ንዚ ትኩር ጉጅለ ከመያይጠክን ወለንታ ብምሃብክን ኣዝየ ኣመስግን፡፡ ዝተወሰኑ ሕቶታት ኣዳልየ ኣለኹ፡፡ ብመቕድሒ ድምፂ ክጥቀም ምኽንየ ከፍልጠክን ይደሊ፡፡ ኣብ'ዚ ከይዲ ትኩር ጉጅለ ምይይጥ ዝኾነ ክትፈልጣአ ወይ ክትርድኣአ ትደልያኦ ብነፃ ምዝራብ ናትክን መሰል ምኧኑ ፈሊጥክን እቲ ትኩር ጉጅለ ምይይጥ ኣብ መንጎ ምዝርራብ ይካኣል እዩ፡፡

ሓበሬታ ትኩር ጉጅለ ተመያየጥቲ ሰራሕተኛታት ጥሙር ጥዕና

ድሕረ ባይታ ሓበሬታ

1.1 ትጉም መለለይ: ___________
1.2 ትጉም ዝተሰርሐሉ ዕለት: ___________
1.3 ትጉም ዝተጀመረሉ ግዘ: ___________
1.4 ትጉም ዝተወደአሉ ግዘ: ___________
1.5 ኮድ ቃለ መሕትት:___________
1.6 ኮድ ቃለ ምይይጥ ፀሓፊ: ___________
1.7 ኮድ ተርጓሚ:___________
1.8 መለለይ ድምፂ ቀዳሕት፡___________

Theme 1: ድሕረ ባይታ ስነ ህዝቢ

መለለይ ተሳተፍቲ	ዝመፃሉ ወረዳ	ዝመፃሉ ጣብያ/ ዝሰርሓሉ ኬላ ጥዕና	ብርኪ ትምህርቲ	ንውሐት ስራሕ ግልጋሎት ብዓመት	መብሪሂ

Section C: **መሳለጥታትን መዐንቐፍትታት ኣፈፃፅማ መደባት ኣመራርሓ ልምዓት ጉጅለ**

ቐንዲ መሕትት	ኣውፃእፅ (መሳለጥታትን መዐንቐፍትታት)
**Theme 2: ኣረዳድኣን ስሚዒትን ግደ ኣመራርሓ ልምዓት ጉጅለ ኣብ ጥዕና**	
2.1 ኣመራርሓ ልምዓት ጉጅለ ደቂ ኣንስትዮ ከም ነባሪቲ ኣብ'ዚ ሕብረተሰብ እንታይ ሓላፍነት ኣለወን?	ካሊእኸ፣ ካሊእኸ ... ካሊእ ኸ... ይቐፅል)
2.2 ኣመራርሓ ልምዓት ጉጅለ ደቂ ኣንስትዮ ስራሕተን ኣብ ክንክን **ጥዕና** ከመይ ከም ዝፍፅማ ክትዛረባና ምኻኣልክን ዶ?<br>2.3 ኣመራርሓ ልምዓት ጉጅለ ደቂ ኣንስትዮ ኣብ ክንክን **ኣዴታት፣ ሓናጡን ህፃናትን** ዘለወን **ስራሕቲን ከይዲ ኣፈፃፅማኦም** ክትዛረባና ምኻኣልክን ዶ?<br>2.4 ፍልይ ኣብልክን ኣብ ክንክን **ሓናጡ** ዘሎ ተግባር ከመይ ይፍፅማኦ ትብላ?	ስዕቦም ዘለዉ መሰረት ብምግባር ውዑይ ትኩር ጉጅለ ምይይጥ ክግበር ኣሳልጥ...?<br>1. ከይዲ ኣተላልማ፣ ኣኸባን ቅብብል ሕሙማት ወይ ፀብፃብ ምስ ሰራሕተኛታት ጥሙር ጥዕና: (ብስልኪ፣ ሕቶን መልስን፣ ኣብ ኩሉ ብርኪታት ኣሳታፉይነት፣ እዋናውነት፣ ካብ ታሕቲ ንላዕሊ፣ ብወረቐት ወይ ብፎርሚ)<br>2. ምትሕብባር ዋዕላ ጥንሳት ኣዴታት: - መድረኽ ዋዕላ ጥንሳት መን ይመርሖ?<br>3. ምይይጥ ኣብ ቅብብል ግልጋሎት ጥኑሳት ኣዴታት ጥዕና:<br>4. ምይይጥ ኣብ ቅብብል ግልጋሎት ንሓራሳት ኣዴታትን ሓደሽቲ ውልዶ ሓናጡን: ኣብ ምንታይ ዛዕባ ትመያየጣ?<br>5. ከመይ ትርእዮ ዑደት ገዛ:<br>6. ምስታፍ ኣብ ጥዕና ተዛመድቲ ወፍርታት፡ ሓደ ን ሓሙሽተ መርበብ ምትሕግጋዝ ልምዓት ጉጅለ፤ ኣብነት፡ ፀረ-ኣልምሲ፣ ትራኮማን ካልኦትን፡፡
መዘኻኸሪ: ምግንዛብ ግደን ስሚዒትን ኣመራርሓ ልምዓት ጉጅለ ደቂ ኣንስትዮ ኣብ ስራሕተንን ውፅኢተን፡፡	
**Thematic 3. ልምዓት ጉጅለ ደቂ ኣንስትዮ ኣብ ክንክን ጥዕና ኣዴታት፣ ሓናጡን ህፃናትን ዘለወን ፍልጠት**	
3.1 ስራሕቲ ምስጓምን ምክልኻልን **ጥዕና** ከም ኣመራርሓ ልምዓት ጉጅለ ደቂ ኣንስትዮ ክህልወን ዝግቦኦ ፍልጠት ኣለወን ዶ ትብላ?<br>3.2 ስራሕቲ ምስጓምን ምክልኻል ጥዕና **ኣዴታት፣ ሓናጡን ህፃናትን** ከም ኣመራርሓ ልምዓት ጉጅለ ደቂ ኣንስትዮ ክህልወን ዝግቦኦ ፍልጠት ኣለወን ዶ ትብላ?<br>3.3 ብፍላይ ክንክን ሓናጡን ሓደገኛ ምልክታት ክህልወን ዝግቦኦ ፍልጠት ኣለወን ዶ ትብላ?<br>3.3 ኣመራርሓ ልምዓት ጉጅለ ደቂ ኣንስትዮ ሓላፍነተን ንኽዋፃኣ እንታይ ዓይነት ክፍተት ፍልጠት እንተተመለአለን ትብላ?	ውዑይ ትኩር ጉጅለ ምይይጥ ክግበር ኣሳልጥ...?
**Thematic 4. ልምዓት ጉጅለ ደቂ ኣንስትዮ ኣብ ክንክን ጥዕና ኣዴታት፣ ሓናጡን ህፃናትን ከይዲ ስልጠና**	
4.1 ናብ ስራሕ መእተዊ ስልጠና ንኣመራርሓ ልምዓት ጉጅለ ደቂ ኣንስትዮ መውህቢ ከይዲ እንተሃልዩ ምገለፅክናልና ዶ?<br>4.2 እቲ ዝዋሃብ ስልጠና ማለት መጀመሪን ተሃድሶን ከይዲ ከመይ ትዕዘባኦ? **ፈላልኻ ክመያየጣሉ ይገበር**<br>4.3 ኣብ ሕሉፍ ዓመት ተሃድሶ ስልጠና እንድሕር ዘይወሰዳ ሃልየን፤ ምኽንያት ክትነግርና ምኻኣልክን ዶ?<br>4.4 ብቲ ዝተውሃበ ወይ ዝዋሃብ ዝነበሩ ስልጠናታት ንኣመራርሓ ልምዓት ጉጅለ ደቂ ኣንስትዮ ኣብ ኣዴታት፣ ሓናጡን ህፃናትን ዝምልከት ንስኻትክን ኣገባቡ ዝተማለአን እኹል እዋን ተዋሂቡዎ ዝብሉን ሓዊስክን ብኸመይ ትገልፃኦ?	ብዛዕባ ከይዲ ኣፈፃፅማ ስልጠናን ውፅኢቶምን መሰረት ብምግባር ውዑይ ትኩር ጉጅለ ምይይጥ ክግበር ኣሳልጥ...?
**Thematic 5. ልምዓት ጉጅለ ደቂ ኣንስትዮ ኣብ ክንክን ጥዕና ኣዴታት፣ ሓናጡን ህፃናትን ከይዲ ሓጋዚ ዑደት**	

5.1 ኣፈፃፅማ ሓጋዛይ ዑደት ሰራሕተኛታት ጥሙር ጥዕና ወይ ካሊእ ኣድላይ ኩነት ኣብ ምምላእን ከይዲን ብኸመይ ትሪኣኦ?<br>5.2 ብኣኻትክን ማለት ብሰራሕተኛታት ጥሙር ጥዕና ዝዋሃበ ሓጋዛይ ዑደት ከይዱ፣ ወጥነቱን ውፅኢቱን እንታይ ሪኢቶ ኣለክን?<br>5.3 ብኣመራርሓ ልምዓት ጉጅለ ደቂ ኣንስትዮ ዝዋሃበ ሓጋዛይ ዑደት ንኣባላት ከይዱ፣ ወጥነቱን ውፅኢቱን እንታይ ሪኢቶ ኣለክን?	ብዛዕባ ከይዲ ኣፈፃፅማ ሓጋዛይ ዑደትን ውፅኢቶምን መሰረት ብምግባር ሜላ ምውፅእፃእ ብዕሙቕት ተጠቐም...?
**Thematic 6. ልምዓት ጉጅለ ደቂ ኣንስትዮ ኣብ ክንክን ጥዕና ኣዴታት፣ ሓናጡን ህፃናትን ከይዲ ምትብባዕን ወይ ኣፍልጦ ምሃብ**	
6.1 እቲ መተባብዒ ዝዋሃብ ንሓፈሻዊ ኣፈፃፅማ ጥዕና ድዩ? ብመነፅር ስርዓት ጥዕናን ሕብረተሰብን<br>6.2 እዙይ ኣብ ጥዕና ኣዴታት፣ ሓናጡን ህፃናትን ብኸመይ ይፍፀም?<br>6.3 ኣፈፃፅማ መተባቢዒ ወይ ኣፍልጦ ኣዋሃህባ ኣብ ከይዲ ኣገባቡ ፍትሓውነቱን ብኸመይ ትሪኣኦ?	ኣውፃእፅእ (ብመነፀር: ፍትሓውነቱ፣ መትከላት፣ ኣሳታፋይነት፣ ግልፅነት፣ ተኣማንነትን ካልኦትን)
**Thematic 7. ልምዓት ጉጅለ ደቂ ኣንስትዮ ኣብ ክንክን ጥዕና ኣዴታት፣ ሓናጡን ህፃናትን ንስራሐን ምቹ ዝኾኑ ከባቢን ኣወዳድባን**	
7.1 ኣብ ዲሞክራሲያዊ ኣወዳድባ ልምዓት ጉጅለ እንኮላይ ኣብ ተዋረድ ሓላፍነት ዘለክን ሪኢቶ ምገለፅክናልና ዶ? ብኣውንታን ብኣሉታን ዘለዎ ፅልዋ<br>7.2 ብኣኻትክን ማለት ሰራሕተኛታት ጥሙር ጥዕና ወይ ብኻሊእ ኣካል ኣመራርሓ መርበብ ምትሕግጋዝ ልምዓት ጉጅለ (ሓደ ን ሓሙሽት) ብማዕረ ከም እተን ሓደ ን30 ኣመራርሓ/ ደገፍ ይረኽባ ዶ እልክን ትኣምና?	ብዛዕባ ዲሞክራሲያዊ ውክልናን ውፅኢቶምን መሰረት ብምግባር ሜላ ምውፅእፃእ ብዕሙቕት ተጠቐም...?
**Thematic 8. ኣመራርሓ ልምዓት ጉጅለ ደቂ ኣንስትዮ ኣብ ክንክን ጥዕና ኣዴታት፣ ሓናጡን ህፃናትን ስራሕቲ ኣሚኒታ ኣብ ኩለን ኣባላትን ካልኦት የእትወና ባሃልትን**	
8.1 ኣመራርሓ ልምዓት ጉጅለ ደቂ ኣንስትዮ ኣብ ሕብረተሰብ፣ ኣባላተን፣ ኣብ ባዕለን ኣመራርሓ ልምዓት ጉጅለ ውሺጢ፣ ደቂ ኣነስትዮ ኣመራርሓን ካልኦትን ዘለወን ኣሚኒታ ብኸመይ ትገልጻኦ?	ውፅኢቶም ብኽልቲኡ ገፅ መሰረት ብምግባር ሜላ ምውፅእፃእ ብዕሙቕት ተጠቐም...?
**Thematic 9. ልምዓት ጉጅለ ደቂ ኣንስትዮ ኣብ ክንክን ጥዕና ኣዴታት፣ ሓናጡን ህፃናትን ዘለወን ርክብ ኣብ መንጎኣንን ምስ ካልኦት ዝተፈላለዩ የእትወና ባሃልቲ**	
9.1 ኣፈፃፅማ ስራሕቲ ኣመራርሓ ልምዓት ጉጅለ ንኽመሓየሽ መሳለጥቲ ወይ መዐንቀፍቲ ብብርኪ ሕብረተሰብን መንግስትን ንገለፅክናል ዶ?<br>9.2 ሜላ ርክብ ኣመራርሓ ልምዓት ጉጅለ ምስ የእትወና ባሃልቲ ኣብ **ክንክን ጥዕና ኣዴታት፣ ሓናጡን ህፃናትን** ዝፈጠሮ ብኣውንታ/ ብኣሉታ ንገለፅክናል ዶ?	ውፅኢቶም ብኽልቲኡ ገፅ መሰረት ብምግባር ሜላ ምውፅእፃእ ብዕሙቕት ተጠቐም...?
ብሓፈሽኡ ኣፈፃፅማ ስራሕቲ ኣመራርሓ ልምዓት ጉጅለ ደቂ ኣንስትዮ ኣብ ሓፈሻዊ ክንክን ጥዕና ብፍላይ ኣብ **ክንክን ጥዕና ኣዴታት፣ ሓናጡን ህፃናትን** ከመይ ትሪኣኦ?	ሜላ ምውፅእፃእ ብዕሙቕት ተጠቐም...?
ብምጥንኻር ኣፈፃፅማ ልምዓት ጉጅለ፤ ሕብረተሰብ ብቁልጥፍን ብቐፃላይን ውፅኢት ኣብ ክንክን ጥዕና ኣዴታት፣ ሓናጡን ህፃናትን ከረጋግፅ እንታይ ተተገበረ ትብላ?	ሜላ ምውፅእፃእ ብዕሙቕት ተጠቐም...?

መዘኻኸሪ: ኣብዙይ ከግለፅን ክምመዩን ዝተደለዩ ኣብ ተግባር መጎልበትን መዐንቀፍትን ዝኾኑ **ንስልጠና፣ ሓጋዚ ዑደት፣ ፍልጠት፣ ኣፍልጦ ምሃብን ንስራሕ ምቹ ዝኾኑ ከባቢን ኣወዳድባን** ክህሉ ኣብ ምግባር ምዝርዛር፡፡ ብተወሳኺ ምንፅፃር ዝተዳለወ ኢደ መጋብርን ብተግባር ዝፍፀም ዘሎን ትፅቢት ትገብረሉ፡፡

መወዳእታ እዙይ መሕትት

ነመስግን፡፡

ርእሲ መፅናዒቲዕ: መሳለጥትን መዐንቀፍትን ንኣመራርሓ ልምዓት ጉጅለ ደቂ ኣንስትዮ ኣብ ምምሕያሽ ኣፈፃፅማ ክንክን ኣዴታት፣ ሓናጡን ህፃናትን፤ ትግራይ፤ ኢትዮጵያ 2010ዓ.ም

ትኩር ጉጅለ ምይይጥ /ትጉም/: መፅናዒቲ ንኣመራርሓ ልምዓት ጉጅለ ደቂ ኣንስትዮ

መእተዊ

ንተ ወረቐት ወለንታ ኣሚንክናሉ ምምላእኺን ንዚ ትኩር ጉጅለ ከመያይጠክን ወለንታ ብምሃብክን ኣዝየ ኣመስግን፡፡ ዝተወሰኑ ሕቶታት ኣዳልየ ኣለኹ፡፡ ብመቕድሒ ድምፂ ክጥቀም ምኺንይ ከፍልጠክን ይደሊ፡፡ ኣብ'ዚ ከይዲ ትኩር ጉጅለ ምይይጥ ዝኾነ ክትፈልጣኦ ወይ ክትርድኣኦ ትደልያኦ ብነፃ ምዝራብ ናትክን መሰል ምኺኑ ፈሊጥክን እቲ ትኩር ጉጅለ ምይይጥ ኣብ መንጎ ምዝርራብ ይካኣል እዩ፡፡

ሓበሬታ ተመያየጥቲ ኣባላት ጉጅለ

ድሕረ ባይታ ሓበሬታ

1.9 ትጉም መለለይ: ______________

1.10 ትጉም ዝተሰርሓሉ ዕለት: ___________

1.11 ትጉም ዝተጀመረሉ ግዘ: ____________

1.12 ትጉም ዝተወደኣሉ ግዘ: ____________

1.13 ኮድ ቃለ መሕትት:_____________

1.14 ኮድ ቃለ ምይይጥ ፀሓፊ: ___________

1.15 ኮድ ተርጓሚ:_____________

1.16 መለለይ ድምፂ ቀዳሕት:____________

Theme 1: ድሕረ ባይታ ስነ ህዝቢ

መለለይ ተሳተፍቲ	ዕድመ	ባዓልቲ ሓዳር ወይ ካሊእ	ብርኪ ትምህርቲ	በዝሒ ኣብ ትሕቲኣ ዘለዋ ኣዴታት	ሓላፍነት (1ን30 ን/ወይ 1ን5)	ኣብ ኣመራርሓ ዝፀንሓትሉ ብዓመት

Section C**: መሳለጥታትን መዐንቐፍትታት ኣፈፃፅማ መደባት ኣመራርሓ ልምዓት ጉጅለ**

ቐንዲ መሕትት	ኣውፃእፅ (መሳለጥታትን መዐንቐፍትታት)
**Theme 2: ኣረዳድኣን ስሚዒትን ግደ ኣመራርሓ ልምዓት ጉጅለ ኣብ ጥዕና**	
2.1 ከም ነበርቲ ኣብ'ዚ ሕብረተሰብ እንታይ ሓላፍነት ኣለክን?	ካሊእኸ፣ ካሊእኸ ... ካሊእ ኸ... ይቕፅል)
2.2 ከም ኣመራርሓ ልምዓት ጉጅለ ደቂ ኣንስትዮ ስራሕቲኺ ኣብ ክንክን **ጥዕና** ክትዝርዝርለይ ምኻኣልክን ዶ?<br>2.3 ኣብ ክንክን **ኣዴታት፣ ሓናጡን ህፃናትን** ዘለዉ **ስራሕቲን ከይዲ ኣፈፃፅማኦም** ክትዛረብኒ ምኻኣልክን ዶ?<br>2.4	ውዑይ ትኩር ጉጅለ ምይይጥ ክግበር ኣሳልጥ...?<br>1. ከይዲ ኣተላልማ፣ ኣኸባን ቅብብል ሕሙማት ወይ ፀብፃብ ምስ ሰራሕተኛታት ጥሙር ጥዕና: (ብስልኪ፣ ሕቶን መልስን፣ ኣብ ኩሉ ብርኪታት ኣሳታፉይነት፣ እዋናውነት፣ ካብ ታሕቲ ንላዕሊ፣ ብወረቐት ወይ ብፎርሚ)<br>2. ምትሕብባር ዋዕላ ጥንሳት ኣዴታት: - መድረኽ ዋዕላ ጥንሳት መን ይመርሖ?<br>3. ምይይጥ ኣብ ቅብብል ግልጋሎት ጥኑሳት ኣዴታት ጥዕና:<br>4. ምይይጥ ኣብ ቅብብል ግልጋሎት ንሓራሳት ኣዴታትን ሓደሽቲ ውልዶ ሓናጡን: ኣብ ምንታይ ዛዕባ ትመያየጣ?<br>5. ከመይ ትርእዮ ዑደት ገዛ :<br>6. ምስታፍ ኣብ ጥዕና ተዛመድቲ ወፍርታት:
**መዘኻኸሪ:** ምግንዛብ ግደን ስሚዒትን ኣመራርሓ ልምዓት ጉጅለ ደቂ ኣንስትዮ ኣብ ስራሕተንን ውፅኢተን::	
**Thematic 3. ልምዓት ጉጅለ ደቂ ኣንስትዮ ኣብ ክንክን ጥዕና ኣዴታት፣ ሓናጡን ህፃናትን ዘለወን ፍልጠት**	
3.1 ስራሕቲ ምስጓምን ምክልኻልን **ጥዕና** ከም ኣመራርሓ ልምዓት ጉጅለ ደቂ ኣንስትዮ ክህልወና ዝግባእ/ ዘፍፅም ፍልጠት ኣለና ዶ ትብላ?<br>3.2 ስራሕቲ ምስጓምን ምክልኻል ጥዕና **ኣዴታት፣ ሓናጡን ህፃናትን** ከም ኣመራርሓ ልምዓት ጉጅለ ደቂ ኣንስትዮ ክህልወና ዝግባእ/ ዘፍፅም ፍልጠት ኣለና ዶ ትብላ?<br>3.3 ኣብ ክንክን ሓናጡ ብፉሉይ ዝግባእ ፍልጠት ኣለና ዶ ትብላ?<br>3.3 ሓላፍነትክን ንኸትዋፀኣ እንታይ ዓይነት ክፍተት ፍልጠት የዕንቐፈክን ትብላ?	ውዑይ ትኩር ጉጅለ ምይይጥ ክግበር ኣሳልጥ...?
**Thematic 4. ልምዓት ጉጅለ ደቂ ኣንስትዮ ኣብ ክንክን ጥዕና ኣዴታት፣ ሓናጡን ህፃናትን ከይዲ ስልጠና**	
4.1 እቲ ዝረኸብክናኦ ስልጠና ከይዲ ከመይ ርእኽናኦ?<br>4.2 ኣብ ሕሉፍ ዓመት ስልጠና እንድሕር ዘይወሰድክን፤ ምኽንያት ክትነግራና ትኽእላ ዶ?<br>4.3 ናብ ስራሕ መእተዊ ስልጠና ከመይ ተገይሩ ይዋሃብ?	ውዑይ ትኩር ጉጅለ ምይይጥ ክግበር ኣሳልጥ...?
**Thematic 5. ልምዓት ጉጅለ ደቂ ኣንስትዮ ኣብ ክንክን ጥዕና ኣዴታት፣ ሓናጡን ህፃናትን ከይዲ ሓጋዚ ዑደት**	
5.1 ኣፈፃፅማ ሓጋዛይ ዑደት ብኸመይ ትሪኣኦ?<br>5.2 ብሰራሕተኛታት ጥሙር ጥዕና ዝዋሃበ ሓጋዛይ ዑደት እንታይ ሪኢቶ ኣለክን?	ውዑይ ትኩር ጉጅለ ምይይጥ ክግበር ኣሳልጥ...?
**Thematic 6. ልምዓት ጉጅለ ደቂ ኣንስትዮ ኣብ ክንክን ጥዕና ኣዴታት፣ ሓናጡን ህፃናትን ከይዲ ምትብባዕን ወይ ኣፍልጦ ምሃብ**	
6.1 እቲ መተባብዒ ዝዋሃብ ንሓፈሻዊ ኣፈፃፅማ ጥዕና ድዩ? ብመነፅር ስርዓት ጥዕናን ሕብረተሰብን<br>6.2 እዙይ ኣብ ጥዕና ኣዴታት፣ ሓናጡን ህፃናትን ብኸመይ ይፍፀም?	(ብመነፀር: መትከላት፣ ኣሳታፉይነት፣ ግልፅነት፣ ተኣማንነትን ካልኦትን)<br>ውዑይ ትኩር ጉጅለ ምይይጥ ክግበር ኣሳልጥ...?

6.3 ኣፈፃፅማ መተባቢዒ ወይ ኣፍልጦ ኣዋሃህባ ኣብ ከይዲ ኣገባቡ ፍትሓውነቱን ብኸመይ ትረኣኣ?	
**Thematic 7. ልምዓት ጉጅለ ደቂ ኣንስትዮ ኣብ ክንክን ጥዕና ኣዴታትን ሓናጡን ህፃናትን ንስራሐን ምቹ ዝኾኑ ከባቢን ኣወዳድባን**	
7.2 ኣብ ዲሞክራሲያዊ ኣወዳድባ ልምዓት ጉጅለ እንኮላይ ኣብ ተዋረድ ሓላፍነት ዘለክን ሪኢቶ ምገለፅክናለይ ዶ? ብኣውንታን ብኣሉታን ዘለዎ ፅልዋ<br>7.2 ብሰራሕተኛታት ጥሙር ጥዕና ወይ ብኻሊእ ኣካል ኣመራርሓ መርበብ ምትሕግጋዝ ልምዓት ጉጅለ (ሓደ ን ሓሙሽት) ብማዕረ ከም እተን ሓደ ን30 ኣመራርሓ/ ደገፍ ይረኽባ ዶ እልክን ትኣምና?	ውዑይ ትኩር ጉጅለ ምይይጥ ክግበር ኣሳልጥ…?
**Thematic 8. ኣመራርሓ ልምዓት ጉጅለ ደቂ ኣንስትዮ ኣብ ክንክን ጥዕና ኣዴታትን ሓናጡን ህፃናትን ስራሕቲ ኣሚኒታ ኣብ ኩለን ኣባላትን ካልኦት የእትወና ባሃልትን**	
8.1 ኣመራርሓ ልምዓት ጉጅለ ደቂ ኣንስትዮ ኣብ ሕብረተሰብ፣ ኣባላተን፣ ኣብ ባዕለን ኣመራርሓ ልምዓት ጉጅለ ውሺጢ፣ ደቂ ኣነስትዮ ኣመራርሓን ካልኦትን ዘለክን ኣሚኒታ ብኸመይ ትገልፃኦ?	ውዑይ ትኩር ጉጅለ ምይይጥ ክግበር ኣሳልጥ…?
**Thematic 9. ልምዓት ጉጅለ ደቂ ኣንስትዮ ኣብ ክንክን ጥዕና ኣዴታትን ሓናጡን ህፃናትን ዘለወን ርክብ ኣብ መንግስትን ምስ ካልኦት ዝተፈላለዩ የእትወና ባሃልቲ**	
9.1 ብብርኪ ኣካላት ሕብረተሰብን መንግስቲን መን ምሳኸን ሓገዝ ኣብ ስራሕትኸን ይገብረልኪ?<br>9.2 ኣብ ምንታይ ተግባራት ምሳኸን ኮይኖም ይሰርሑ ወይ ይተሓባበሩ?<br>9.3 ርክብክን ትፅብትክን ኣብ ምዕዋት **ክንክን ጥዕና ኣዴታት፣ ሓናጡን ህፃናትን** ካብ የእትወና ባሃልቲ ምገለፅክናልና ዶ?	ውዑይ ትኩር ጉጅለ ምይይጥ ክግበር ኣሳልጥ…?
ከም ኣመራርሓ ልምዓት ጉጅለ ደቂ ኣንስትዮ ኣፈፃፅማ ስራሕትኸን **ክንክን ጥዕና ኣዴታት፣ ሓናጡን ህፃናትን** ከመይ ትረኣኣ?	ውዑይ ትኩር ጉጅለ ምይይጥ ክግበር ኣሳልጥ…?
ብምጥንኻር ኣፈፃፅማ ልምዓት ጉጅለ፤ ሕብረተሰብ ብቁልጥፍን ብቐፃላይን ውፅኢት ኣብ ክንክን ጥዕና ኣዴታት፣ ሓናጡን ህፃናትን ከረጋግፅ እንታይ ተተገበረ ትብላ?	ውዑይ ትኩር ጉጅለ ምይይጥ ክግበር ኣሳልጥ…?

መዘኻኸሪ: ኣብዙይ ክግለፅን ክምመዩን ዝተደለዩ ኣብ ተግባር መጎልበትን መዐንቀፍትን ዝኾኑ ን**ስልጠና፣ ሓጋዚ ዑደት፣ ፍልጠት፣ ኣፍልጦ ምሃብን ንስራሕ ምቹ ዝኾኑ ከባቢን ኣወዳድባን** ክህሉ ኣብ ምግባር ምዝርዛር፡፡ ብተወሳኺ ምንፅፃር ዝተዳለወ ኢደ መጋብርን ብተግባር ዝፍፀም ዘሎን ትፅቢት ትገብረሉ፡፡

መወዳእታ እዙይ መሕትት

ነመስግን፡፡

ርእሲ መፅናዒቲዕ: መሳለጥትን መዐንቀፍትን ንኣመራርሓ ልምዓት ጉጅለ ደቂ ኣንስትዮ ኣብ ምምሕያሽ ኣፈፃፅማ ክንክን ኣዴታት፣ ሓናጡን ህፃናትን፤ ትግራይ፤ ኢትዮጵያ 2010ዓ.ም

ዓሙቕ መሕትት: መፅናዒቲ ንልምዓት ጉጅለ ደቂ ኣንስትዮ

መእተዊ

ንቲ ወረቐት ወለንታ ኣሚንክሉ ምምላእኺን ንዚ ቃለ መሕትት ክሓተክን ወለንታ ብምሃብኪ ኣዝዩ ኣመስግን፡፡ ዝተወሰኑ ሕቶታት ኣዳልየ ኣለኹ፡፡ ብመቕድሒ ድምፂ ክጥቀም ምኻነይ ከፍልጠኪ ይደሊ፡፡ ኣብዚ ከይዱ ምሕታት ዝኾነ ክትፈልጥዮ ወይ ክትርድእዮ ትደልይዮ ብነፃ ምዝራብ ናትኪ መሰል ምኻኑ ፈሊጥኪ እቲ ምሕታት ኣብ መንጎ ምዝርራብ ይካኣል እዩ፡፡

Theme 1: ክፍሊ ሓደ፤ ዝርዝር ኩነታት መሕትትን ድሕረ ባይታን ስነ ህዝቢን

ክፍሊ ሀ/ ዝርዝር ኩነታት መሕትት

1 ክልል: ______________
2 ዞባ:______________
3 ወረዳ:______________
4 ጣብያ:______________
5 ሽም ዓሙቕ መሕትት ተሓታታይ:______________
6. ሽም ዓሙቕ መሕትት ተሓታታይ ትካል:______________
7. ሽም ዓሙቕ መሕትት ሓታታይ ትካል:______________
8. መሕትት ዝተኻየደሉ ዕለት: ______________
8. መሕትት ዝተጀመረሉ ሰዓት: ______________
9. መሕትት ዝተወደአሉ ሰዓት: ______________

10. ሓታቲ መለለይ: ______________

11. መቅድሒ መሳርሒ ቁፅሪ: ______________

ክፍሊ ለ/ ድሕረ ባይታን ስነ ህዝቢን

1. መለለይ ቁፅሪ : ________________
2. መንበሪ ቦታ : _______________________
2. ኩነታት ሓዳር: ________________
3. ፆታ: ____________
4. ሃይማኖት: _________________
5. ብርኪ ትምህርቲ: ____________
6. ዕድመ ተሳተፍቲ ብዓመት: ______________

7. ንክንደይ ኣዴታት ተተሓባብሪ: ______________
8. ስራሕ ልምዲ ብኣዋርሕ: ______________

Section C: **መሳለጥታትን መዐንቐፍትታት ኣፈፃፅማ መደባት ኣመራርሓ ልምዓት ጉጅለ**

ቐንዲ መሕትት	ኣውፃእፅ (መሳለጥታትን መዐንቐፍትታት)
**Theme 2: ኣረዳድኣን ስሚዒትን ግደ ኣመራርሓ ልምዓት ጉጅለ ኣብ ጥዕና**	
2.1 ከም ነባሪት ኣብዚ ሕብረተሰብ እንታይ ሓላፍነት ኣለኪ? (ካሊእ ኸ... ይቕፅል)	ጥዕና፣ ሕርሻ፣ ማይ፣ ትምህርቲ፣ ፀጥታን ሰናይ ምምሕዳርን ካልኦትን
2.2 ከም ኣመራርሓ ልምዓት ጉጅለ ደቂ ኣንስትዮ ስራሕቲኺ ኣብ ክንክን **ጥዕና** ክትዝርዝርለይ ምኻኣልኪ ዶ?<br>2.3 ኣብ ክንክን **ኣዴታት፣ ሓናጡን ህፃናትን** ዘለዉ **ስራሕቲን ከይዲ ኣፈፃፅማኦም** ክትዛረብኒ ምኻኣልኪ ዶ?<br>2.4	1. ከይዲ ኣተላልማ፣ ኣኸባን ቅብብል ሕሙማት ወይ ፀብፃብ ምስ ሰራሕተኛታት ጥሙር ጥዕና: (ብስልኪ፣ ሕቶን መልስን፣ ኣብ ኩሉ ብርኪታት ኣሳታፋይነት፣ እዋናውነት፣ ካብ ታሕቲ ንላዕሊ፣ ብወረቐት ወይ ብፎርሚ)<br>በቢ ክንደይ ኣፈላላይ ኣኼባ ተካይዳ?<br>• ምስ ኩለን ኣባላት መርበብ ምትሕግጋዝ ልምዓት ጉጅለ (6)______<br>• ምስ ኩለን ኣባላት ልምዓት ጉጅለ (30)_______<br>• ኣመራርሓ መርበብ ምትሕግጋዝ ልምዓት ጉጅለ (5)______<br>• ሓደ ን ሓሙሽተ ምስ ሰራሕተኛታት ጥሙር ጥዕና _____<br>• ሓደ ን ሳላሳ ምስ ሰራሕተኛታት ጥሙር ጥዕና<br>2. ምትሕብባር ዋዕላ ጥንሳት ኣዴታት: - መድረኽ ዋዕላ ጥንሳት መን ይመርሖ?<br>- ከመይ ገርኪ ስራሕትኺ ቁልጥፍ ምፍላይ ጥንሳት ኣዴታት ተካይዲ?<br>- ኩለን ክሳተፋ ዝግበኣን ጥኑሳት ኣዴታት ኣብ ዋዕላ ጥኑሳት ይሳተፋ እየን እክሊ ትሓስቢ ዶ? እንድሕር ዘይኮነ ንምንታይ?<br>- እንታይ ኣጀንደታት ይታሓዙን ምይይጥ ይግበረሎምን?<br>3. ምይይጥ ኣብ ቅብብል ግልጋሎት ጥኑሳት ኣዴታት ጥዕና:<br>- ምስ ጥኑሳት ኣዴታት ክስርሑ ዝግቦኦም ኣስተምህሮ ይካየዱ ዶ? እንድሕር እወ ኮይኑ ኣበይኖት ቀንዲ ኣርኣስቲ ምይይጥ ይግበር:<br>-ረብሓ ብእዋን ቅድመ ወሊድ ክትትል ምጅማርን ትኩር ቅድመ ወሊድ ክትትልን,<br>-ምርመራ ኤችኣይቪን ምምሕያሽ ኣመጋግባን,<br>-ድልውነት: ንዋት ምቁጣብ፣ ዘድልዩ ዓለባታት፣ ባህላዊ ኣምቡላንስ ብምድላው ምጥቃም፣ ጥኑሳት ኣዴታት ስልኪ ኣመራርሓ መርበብ ምትሕግጋዝ ልምዓት ጉጅለታት፣ ሰራሕተኛ ጥሙር ጥዕናን ካልኦት ሰራሕተኛታ ጥዕናን ካልኦትን<br>- ሓደገኛ ምልክታት ጥንሲ? (መድመይቲ ማህፀን፣ ርእሲ ሕማም፣ ፀረውረውታ/ ምርባሽ ኣእምሮ፣ ልዑል ፀቅጢ ደምን ካልኦትን<br>- መዕረፊ ጥኑሳት ኣዴታት<br>4. ምይይጥ ኣብ ቅብብል ግልጋሎት ንሓራሳት ኣዴታትን ሓደሽቲ ውልዶ ሓናጡን: ኣብ ምንታይ ዛዕባ ትመያየጣ?<br>-ክንክንን ድሕረ ወሊድ ክትትልን: ንሓራሳት ኣዴታትን ሓደሽቲ ውልዶ ሓናጡን ,<br>- ሓደገኛ ምልክታት ሓናጡን<br>- ሓደገኛ ምልክታት ህፃናትን<br>5. ከመይ ትርእዮ ዑደት ገዛ :

<table>
<tr><td></td><td>- ዳህሳስ ምፍላይን ቁልጥፉ ኣለሻ ዘይጀመራን ዝጀመራን ጥኑሳት ኣዴታት,
- ክትትልን: ንሓራሳት ኣዴታት፣ ሓደሽቲ ውልዶ ሓናጡን ህፃናትን ከም ክትባት፣ ዕብየት ክትትል፣ ከባቢ ፅሬትን ውልቀ ፅሬትን ካልኦትን
6. ምስታፍ ኣብ ጥዕና ተዛመድቲ ወፍርታት: ሓደ ን ሓሙሽተ መርበብ ምትሕግጋዝ ልምዓት ጉጅለ፤ ኣብነት: ፀረ-ኣልምሲ፣ ትራኮማን ካልኦትን፡፡</td></tr>
<tr><td colspan="2">መዘኻኸሪ: ምግንዛብ ግደን ስሚዒትን ኣመራርሓ ልምዓት ጉጅለ ደቂ ኣንስትዮ ኣብ ስራሕተንን ውፅኢተን፡፡</td></tr>
<tr><td colspan="2">Thematic 3. ልምዓት ጉጅለ ደቂ ኣንስትዮ ኣብ ክንክን ጥዕና ኣዴታት፣ ሓናጡን ህፃናትን ዘለወን ፍልጠት</td></tr>
<tr><td>3.1 ስራሕቲ ምስጓምን ምክልኻልን ጥዕና ከም ኣመራርሓ ልምዓት ጉጅለ ደቂ ኣንስትዮ ክህልወኒ ዝግባእ/ ዘፍፅም ፍልጠት ኣለኒ ዶ ትብሊ?

3.2 ስራሕቲ ምስጓምን ምክልኻል ጥዕና ኣዴታት፣ ሓናጡን ህፃናትን ከም ኣመራርሓ ልምዓት ጉጅለ ደቂ ኣንስትዮ ክህልወኒ ዝግባእ/ ዘፍፅም እኹል ፍልጠት ኣለኒ ዶ ትብሊ?
3.3 ኣብ ጥዕና ሓናጡ ክህልወኒ ዝግባእ እኹል ፍልጠት ኣለኒ ዶ ትብሊ?

3.4 ሓላፍነትኪ ንኽትዋፅእ እንታይ ዓይነት ክፍተት ፍልጠት ብዘይምህላወይ ይውሰን ትብሊ?</td><td>ብዛዕባ ጉዳያት መደባቲኪ ጥዕና ክተብራህርህለይ ዶ ትኽእሊ?
- ሓደገኛ ምልክታት ጥኑሳት
- ክንክን ድሕረ ወሊድ ክትትልን መኣዝን መኣዝን እንታይ ዓይነት ክንክን: ንሓራሳት ኣዴታትን ሓደሽቲ ውልዶ ሓናጡን፤ ,
- ሓደገኛ ምልክታት ሓናጡን
- ሓደገኛ ምልክታት ህፃናትን</td></tr>
<tr><td colspan="2">Thematic 4. ልምዓት ጉጅለ ደቂ ኣንስትዮ ኣብ ክንክን ጥዕና ኣዴታት፣ ሓናጡን ህፃናትን ከይዲ ስልጠና</td></tr>
<tr><td>4.1 እቲ ዝረኸብክዮ ስልጠና ከይዲ ከመይ ርእኽዮ?
4.2 ኣብ ሕሉፍ ዓመት ስልጠና እንድሕር ዘይወሰድኪ፤ ምኽንያት ክትነግርኒ ትኽእሊ ዶ?
4.3 ናብ ስራሕ መእተዊ ስልጠና ከመይ ተገይሩ ተዋሂቡኪ?</td><td>ብዛዕባ ከይዲ ኣፈፃፅማ ስልጠና ዝካየድ ዝነበረ ክተብራህርህለይ ዶ ትኽእሊ?
• ንኽንደይ እዋን ተዋሂቡ /ምድግጋም ፣
• ስሩዕነት፣
• እንታይ እንታይ ዓይነት ኣርእስቲታት ተዋሂቡኪ
• ንውሐት ብመዓልቲ ____ ወይ ብሰዓት ___ ዝወደኦ ትነግርኒ ትኽእሊ ዶ?
• ሰራሕተኛታት ጥሙር ጥዕና መሰልጠኒ ስልጠና ወሲደን ዶ?
• ኢደ መጋቢር /ሽኻል መሰልጠኒ ልምዓት ጉጅለ ትጥቀማ ዶ?
• ንስልጠና ኣብ ዝምቹ ማለት ኣብ ክፍሊ ውሽጥን መቀመጥን፣ ፅሕፈት መሳሪሒን ካልኦትን
• ኣዴታትን፣ ሓናጡን ህፃናትን ዝምልከት ስልጠና ተዋሂቡ ድዩ?
• ብግዝ ሰሌዳ ዝተደገፈ ብቅድመ ተኸተል ንሰራሕተኛታት ጥሙር ጥዕና ዝተውሃበ ድዩ?
• ብቲ ዝተውሃበ ወይ ዝዋሃብ ዝነበሩ ስልጠናታት ኣዴታት፣ ሓናጡን ህፃናትን ዝምልከት ንስኺ ዕግበትኪ ብኸመይ ትገልፅዮ?</td></tr>
<tr><td colspan="2">Thematic 5. ልምዓት ጉጅለ ደቂ ኣንስትዮ ኣብ ክንክን ጥዕና ኣዴታት፣ ሓናጡን ህፃናትን ከይዲ ሓጋዚ ዑደት</td></tr>
<tr><td>5.1 ኣፈፃፅማ ሓጋዛይ ዑደት ብኸመይ ትርእዮ?</td><td>• ኢደ መጋብር/ ሽኻል ኣዴታትን፣ ሓናጡን ህፃናትን ዝሓወሰ ሓጋዛይ ዑደት ንኣመራርሓ ልምዓት ጉጅለ ሪኢኺ ዶ ነርኪ?
• ሰራሕተኛታት ጥሙር ጥዕና ወይ ሓጋዛይ ዑደት ትገብር ኣካል ቅድምያ ክእለት ሓጋዛይ ዑደት ስልጠና ዝረኸበት ድያ?</td></tr>
</table>

<table>
<tr><td>5.2 ብሰራሕተኛታት ጥሙር ጥዕና ዝዋሃበ ሓጋዛይ ዑደት እንታይ ሪኢቶ ኣለኪ?</td><td>• ሰራሕተኛታት ጥሙር ጥዕና ወይ ሓጋዛይ ዑደት ትገብር ኣካል ፍልጠት ኣብ ተካይዶ ሓጋዛይ ዑደት ኣለዋ እልኪ ትኣሚኒ ዶ?
• ድግግም ሓጋዛይ ዑደት ክንደይ እዩ ትብሊ? ኣብ ሕሉፍ ሕዱሽተ ኣዋርሕ
• ሕፅረት መሳርሒ ሓጋዛይ ዑደት
• ሓጋዛይ ዑደት ዝገብር ኣካል ሓጋዛይ መሕትት ይጥቀማ ዶ?
• ግብረ መልሲ/ ዳግመ ቅለባ ንመን ይዋሃብ? ንኣመራርሓ ልምዓት ጉጅለ፤ ንምምሕድዳር፣ ጉዳይ ደቂ ኣንስትዮ፣ ጥምረት ደቂ ኣንስትዮን ካልኦትን</td></tr>
<tr><td colspan="2">Thematic 6. ልምዓት ጉጅለ ደቂ ኣንስትዮ ኣብ ክንክን ጥዕና ኣዴታት፣ ሓናጡን ህፃናትን ከይዲ ምትብባዕን ወይ ኣፍልጦ ምሃብ</td></tr>
<tr><td>6.1 ኣፈፃፅማ መተባቢዒ ወይ ኣፍልጦ ኣዋሃህባ ኣብ ከይዲ ኣገባቡ ፍትሓውነቱን ብኸመይ ትርእዮ? ካብ ሓፈሻዊ ሐዚ ትፍፅሞም ተግባራት
6.2 እቲ መተባብዒ ዝዋሃብ ንሓፈሻዊ ኣፈፃፅማ ጥዕና ድዩ? ብመነፅር ስርዓት ጥዕናን ሕብረተሰብን
6.3 እዙይ ኣብ ጥዕና ኣዴታት፣ ሓናጡን ህፃናትን ብኸመይ ይፍፀም?</td><td>(ብመነፀር: መትከላት፣ ኣሳታፉይነት፣ ግልፅነት፣ ተኣማንነትን ካልኦትን)
• ስርዓት ጥዕና: ብሰራሕተኛታት ጥሙር ጥዕና, ጣብያ ጥዕና፣ ወረዳ፣ ክልል፣ ፌደራል ሓለዋ ጥዕና፣ ገበርቲ ሰናይን ካልኦትን
• ስርዓት ሕብረተሰብ: ንምምሕድዳር/ ኩማንድ ፖስት፣ ጉዳይ ፣ ስትሪንግ ደቂ ኣንስትዮ፣ ጥምረት ደቂ ኣንስትዮን፣ ውልቀ ኣባል ነባሪ፣ እድራትን ካልኦት የእትወና ባሃልቲን</td></tr>
<tr><td colspan="2">Thematic 7. ልምዓት ጉጅለ ደቂ ኣንስትዮ ኣብ ክንክን ጥዕና ኣዴታት፣ ሓናጡን ህፃናትን ንስራሖን ምቹ ዝኾኑ ከባቢን ኣወዳድባን</td></tr>
<tr><td>7.3 ኣብ ኣወዳድባ ልምዓት ጉጅለ እንኮላይ ተዋረድ ሓላፍነት ዘለኪ ሪኢቶ ምገለፅክለይ ዶ?

7.2 ኣመራርሓ መርበብ ምትሕግጋዝ ልምዓት ጉጅለ ብሰራሕተኛታት ጥሙር ጥዕና ወይ ብኻሊእ ኣካል ግቡእ ኣመራርሓ/ ደገፍ ይረኽባ/ ንረክብ ዶ ትብሊ?</td><td>• መጠነ ኣመራርሓ መርበብ ምትሕግጋዝ ልምዓት ጉጅለ ምስ በዝሒ ዝተቆፀራ ሰራሕተኛታት ጥሙር ጥዕና ይሳነ/ ሚዛናዊ ዶ ትብሊ? ምላሽኪ እወ እንተኾይኑ
• ኣብ ኣመራርሓ መርበብ ምትሕግጋዝ ልምዓት ጉጅለ ምስ በዝሒ ዝተቆፀራ ሰራሕተኛታት ጥሙር ጥዕና ዘይሚዛናዊ/ ዘይሳነ እንተልኪ እንታይ ሓገዝ ወይ ደገፍ ይተርፍ ወይ ይውሕድ ይመስለኪ? (ሓጋዛይ ዑደት፣ ስልጠና፣ ኣፍልጦን ካልኦትን)
• ሐዚ ኣብ ተግባር ዘሎ ኣወዳድባን ተዋረድ ሓላፍነትን ኣመራርሓ መርበብ ምትሕግጋዝ ልምዓት ጉጅለ ምስ ሰራሕተኛታት ጥሙር ጥዕና ምገለፅክለይ ዶ?
• ከይዲ ኣመራርፃ ኣመራርሓ ልምዓት ጉጅለ ምገለፅክለይ ዶ?
  ○ መርኣያ/ ሞዴል ስድራቤት ዝተመረቐት
  ○ ንጡፍ ተሳታፊ/ት ኣብ እዋን መረፃ ኣመራርሓ ልምዓት ጉጅለ መን እያ?
  ○ ንመረፅቲ ነፃነት /ዲሞክራሲ ይረጋገፅ ዶ?
  ○ ገለ ኣካላት ወይ ውልቀ ሰባት መበገሲ ሕዞም ይመፁ ድዮም ማለት እዚኣ ተተኾውን ዝብል
  ○ ገለ ኣካላት ወይ ውልቀ ሰባት ባዕለን/ ባዕሎም እዚኣ መራሒትክን እያ እለን ድዩን ዝመርፃ
• ከይዲ ኣወራርዳ ኣመራርሓ ልምዓት ጉጅለ ምገለፅክለይ ዶ?</td></tr>
</table>

	○ ንጡፍ ተሳታፊ/ት ኣብ እዋን ምውራድ ኣመራርሓ ልምዓት ጉጅለ መን እያ?<br>○ ኣብ ምውራድ ኣባላት ብነፃነት /ዲሞክራሲ ይረጋገፅ ዶ?<br>○ ገለ ኣካላት ወይ ውልቀ ሰባት መበገሲ ሕዞም ይመፁ ድዮም ንምውራድ ማለት እዚኣ ተትወርድ ዝብል<br>○ ገለ ኣካላት ወይ ውልቀ ሰባት ባዕለን/ ባዕሎም እዚኣ ካብ ሐዚ ንድሓር ወሪወይ ወሪድኪ እኺ እለን ድየን ዝመርፃ
**Thematic 8. ኣመራርሓ ልምዓት ጉጅለ ደቂ ኣንስትዮ ኣብ ክንክን ጥዕና ኣዴታት፣ ሓናጡን ህፃናትን ስራሕቲ ኣሚኒታ ኣብ ኩለን ኣባላትን ካልኦት የእትወና ባሃልትን**	
8.1 **ኣመራርሓ ልምዓት ጉጅለ ደቂ ኣንስትዮ ኣብ ሕብረተሰብ ዘለክን ኣሚኒታ ብኸመይ ትገልፅዮ**?	ተመኩረኺ ዶ ክትነግርኒ ምኻአልኪ?<br>ፍልፍል መረዳእታ ካበይ ትረኽብዮ?<br>ኣብ መንጎ ኣባላትክን ዘለክን ስምምዕነት?<br>ኣብ መንጎ ኣመራርሓ መርበብ ምትሕግጋዝ ልምዓት ጉጅለታት ዘለክን ስምምዕነት?<br>ኣብ መንጎ ኣመራርሓ ሓደ ን30 ልምዓት ጉጅለታት ዘለክን ስምምዕነት?
**Thematic 9. ልምዓት ጉጅለ ደቂ ኣንስትዮ ኣብ ክንክን ጥዕና ኣዴታት፣ ሓናጡን ህፃናትን ዘለወን ርክብ ኣብ መንጎኣንን ምስ ካልኦት ዝተፈላለዩ የእትወና ባሃልቲ**	
9.1 መን ምሳኺ ሓገዝ ኣብ ስራሕትኺ ይገብረልኪ?<br>9.2 ኣብ ምንታይ ተግባራት ምሳኺ ኮይኖም ይሰርሑ ወይ ይተሓባበሩ?<br>9.3 ርክብክን ትፅብትክን ኣብ ምዕዋት **ክንክን ጥዕና ኣዴታት፣ ሓናጡን ህፃናትን** ካብ የእትወና ባሃልቲ ምገለፅክለይ ዶ?	**ብብርኺ ሕብረተሰብ** (ሕብረተሰብ ባህሊ፣ሃይማኖታዊ ስርዓት ሰበብ ገይሮምን መራሕቲ ሃይማኖትን፣ ተፅእኖ ፈጠርቲ ሰባት፣ ማሕበር፣ ስትሪንግ ደቂ ኣንስትዮ፣ ጥምረት ደቂ ኣንስትዮን፣ ደቂ ተባዕትዮ ልምዓት ጉጅለ፣ ሓረስቶት ማሕበር፣ መናእሰይ ማሕበር ወይ ዘይተጠርነፉ ውልቀ መናእሰይ፣ እድራት፣ ባህላዊ ሓኻይም፣ ልምዲ መዋልዳን ካልኦትን<br>**ብብርኺ መንግስቲ** (ስርዓት ጥዕና፡ ሰራሕተኛታት ጥሙር ጥዕና፣ ሱፐርቫይዘር ጥሙር ጥዕና፣ ጣብያ ጥዕና፣ ወረዳ ጥዕና፣ ንምምሕድዳር/ ኩማንድ ፖስት፣ ጉዳይ፣ ጉዳይ ደቂ ኣንስትዮ፣ ትምህርቲ፣ ሕርሻ፣ ማይ፣ ስልጠናን ካልኦትን፡፡
ከም ኣመራርሓ ልምዓት ጉጅለ ደቂ ኣንስትዮ ኣፈፃፅማ ስራሕትኺ **ክንክን ጥዕና ኣዴታት፣ ሓናጡን ህፃናትን** ከመይ ትርእዮ?	• ኣፈፃፅማ ስራሕትኺ እንታይ ትብልዮ? ትሑት/ ማእኸላይ/ ልዑል<br>• ብኣፈፃፅማኺ ብመነፀር ትልሚኺ ዕግብቲ ድኺ?<br>• ንኣፈፃፅማ ስራሕትኺ ከመሓይሹለይ ትብልዮም ምገለፅክለይ ዶ?
ኣፈፃፅማ ልምዓት ጉጅለ ብምጥንኻር ሕብረተሰብ ብቁልጥፍን ብቐፃላይን ውፅኢት ኣብ ክንክን ጥዕና ኣዴታት፣ ሓናጡን ህፃናትን ከረጋግፅ እንታይ ተተገበረ ትብሊ?	ሜላ ምውፅእፃእ ብዕሙቐት ተጠቐም...?

መዘኻኸሪ: ኣብዙይ ክግለፅን ክምመዩን ዝተደለዩ ኣብ ተግባር መጎልበትን መዐንቀፍትን ዝኾኑ ን**ስልጠና፣ ሓጋዚ ዑደት፣ ፍልጠት፣ ኣፍልጦ ምሃብን ንስራሕ ምቹ ዝኾኑ ከባቢን ኣወዳድባን** ክህሉ ኣብ ምግባር ምዝርዛር፡፡ ብተወሳኺ ምንፅፃር ዝተዳለወ ኢደ መጋብርን ብተግባር ዝፍፀም ዘሎን ትፅቢት ትገብረሉ፡፡

መወዳእታ እዙይ መሕትት

ነመስግን፡፡

ርእሲ መፅናዒቲዕ፡ መሳለጥትን መዐንቀፍትን ንኣመራርሓ ልምዓት ጉጅለ ደቂ ኣንስትዮ ኣብ ምምሕያሽ ኣፈፃፅማ ክንክን ኣዴታት፣ ሓናጡን ህፃናትን፤ ትግራይ፤ ኢትዮጵያ 2010ዓ.ም

ዓሙቕ መሕትት፡ መፅናዒቲ ንክሊላታት ጥዕና ወረዳ፤ ክልል

መእተዊ

ንቲ ወረቐት ወለንታ ኣሚንክሉ ምምላእኺን ንዚ ቃለ መሕትት ክሓተክን ወለንታ ብምሃብኪ ኣዝዩ ኣመስግን። ዝተወሰኑ ሕቶታት ኣዳልየ ኣለኹ። ብመቕድሒ ድምፂ ክጥቀም ምኽነይ ክፍልጠኪ ይደሊ። ኣብዚ ከይዲ ምሕታት ዝኾነ ክትፈልጥዮ ወይ ክትርድእዮ ትደልይዮ ብነፃ ምዝራብ ናትኪ መሰል ምኻኑ ፈሊጥኪ እቲ ምሕታት ኣብ መንጎ ምዝርራብ ይካኣል እዩ።

Theme 1: ክፍሊ ሓደ፤ ዝርዝር ኩነታት መሕትትን ድሕረ ባይታን ስነ ህዝቢን

ክፍሊ ሀ/ ዝርዝር ኩነታት መሕትት

1 ክልል: ______________
2 ዞባ:______________
3 ወረዳ:______________
4 ጣብያ:______________
5 ሽም ዓሙቕ መሕትት ተሓታታይ:______________
6. ሽም ዓሙቕ መሕትት ተሓታታይ ትካሉ:______________
7. ሽም ዓሙቕ መሕትት ሓታታይ ትካል:______________
8. መሕትት ዝተኻየደሉ ዕለት: ______________
8. መሕትት ዝተጀመረሉ ሰዓት: ______________
9. መሕትት ዝተወደአሉ ሰዓት: ______________
10. ሓታቲ መለለይ: ______________
11. መቕድሒ መሳርሒ ቁፅሪ: ______________

ክፍሊ ለ/ ድሕረ ባይታን ስነ ህዝቢን

1. መለለይ ቁፅሪ : ________________
2. መንበሪ ቦታ : _______________________
2. ኩነታት ሓዳር: ________________
3. ፆታ: ____________
4. ሃይማኖት: _________________
5. ብርኪ ትምህርቲ: ____________
6. ዕድመ ተሳታፊ/ት ብዓመት: ______________
7. ስራሕን ሓላፍነትን: ______________
8. ስራሕ ልምዲ ብዓመት: ______________

Section C: **መሳለጥታትን መዐንቐፍትታት ኣፈፃፅማ መደባት ኣመራርሓ ልምዓት ጉጅለ**

ቐንዲ መሕትት	ኣውፃኢፅ (መሳለጥታትን መዐንቐፍትታት)
**Theme 2: ኣረዳድኣን ስሚዒትን ግደ ኣመራርሓ ልምዓት ጉጅለ ኣብ ጥዕና**	
2.1 ኣመራርሓ ልምዓት ጉጅለ ደቂ ኣንስትዮ ከም ነባሪቲ ኣብ'ዚ ሕብረተሰብ እንታይ ሓላፍነት ኣለወን?	ካሊእ'ኸ፣ ካሊእ'ኸ ... ካሊእ ኸ... ይቕፅል)
2.2 ኣመራርሓ ልምዓት ጉጅለ ደቂ ኣንስትዮ ስራሕተን ኣብ ክንክን **ጥዕና** ከመይ ከም ዝፍፅማ ክትዛረብኒ/ኒ ምኻኣልኪ/ ምኻኣልካ ዶ?<br>2.3 ኣመራርሓ ልምዓት ጉጅለ ደቂ ኣንስትዮ ኣብ ክንክን **ኣዴታት፣ ሓናጡን ህፃናትን** ዘለወን **ስራሕቲን ከይዲ ኣፈፃፅማኦም** ክትዛረብኒ/ ክትዛረበኒ ምኻኣልኪ/ ምኻኣልካ ዶ?<br>2.4 ፍልይ ኣብልኪ/ ኣብልካ ኣብ ክንክን **ሓናጡ** ዘሎ ተግባር ከመይ ይፍፅማኦ ትብለሐ ትብል?	1. ከይዲ ኣተላልማ፣ ኣኼባን ቅብብል ሕሙማት ወይ ፀብፃብ ምስ ሰራሕተኛታት ጥሙር ጥዕና: (ብስልኪ፣ ሕቶን መልስን፣ ኣብ ኩሉ ብርኪታት ኣሳታፋይነት፣ እዋናውነት፣ ካብ ታሕቲ ንላዕሊ፣ ብወረቐት ወይ ብፎርሚ)<br>2. ምትሕብባር ዋዕላ ጥንሳት ኣዴታት: - መድረኽ ዋዕላ ጥንሳት መን ይመርሖ?<br>- ከመይ ገይረን ስራሕተን ቁልጥፍ ምፍላይ ጥንሳት ኣዴታት የካይዳ?<br>- ኩለን ክሳተፋ ዝግበአን ጥኑሳት ኣዴታት ኣብ ዋዕላ ጥኑሳት ይሳተፋ እየን እልኪ/ እልካ ተሓስብ/ ትሓስቢ ዶ? እንድሕር ዘይኮነ ንምንታይ?<br>- እንታይ ኣጀንደታት ይታሓዙን ምይይጥ ይግበረሎምን?<br>3. ምይይጥ ኣብ ቅብብል ግልጋሎት ጥኑሳት ኣዴታት ጥዕና:<br>4. ምይይጥ ኣብ ቅብብል ግልጋሎት ንሓራሳት ኣዴታትን ሓደሽቲ ውልዶ ሓናጡን: ኣብ ምንታይ ዛዕባ ትመያየጣ?<br>5. ከመይ ትርእዮ ዑደት ገዛ:<br>6. ምስታፍ ኣብ ጥዕና ተዛመድቲ ወፍርታት: ሓደ ን ሓሙሽተ መርበብ ምትሕግጋዝ ልምዓት ጉጅለ፤ ኣብነት: ፀረ-ኣልምሲ፣ ትራኮማን ካልኦትን::
**መዘኻኸሪ:** ምግንዛብ ግደን ስሚዒትን ኣመራርሓ ልምዓት ጉጅለ ደቂ ኣንስትዮ ኣብ ስራሕተንን ውፅኢተን::	
**Thematic 3. ልምዓት ጉጅለ ደቂ ኣንስትዮ ኣብ ክንክን ጥዕና ኣዴታት፣ ሓናጡን ህፃናትን ዘለወን ፍልጠት**	
3.1 ስራሕቲ ምስጓምን ምክልኻልን **ጥዕና** ከም ኣመራርሓ ልምዓት ጉጅለ ደቂ ኣንስትዮ ክህልወን ዝግባእ/ ዘፍፅም ፍልጠት ኣለወን ዶ ትብሊ/ ትብል?<br>3.2 ስራሕቲ ምስጓምን ምክልኻል ጥዕና **ኣዴታት፣ ሓናጡን ህፃናትን** ከም ኣመራርሓ ልምዓት ጉጅለ ደቂ ኣንስትዮ ክህልወን ዝግባእ/ ዘፍፅም እኹል ፍልጠት ኣለወን ዶ ትብሊ/ ትብል?<br>3.3 ብፍላይ ክንክን ሓናጡን ሓደገኛ ምልክታት ክህልወን ዝግባእ ፍልጠት ኣለወን ዶ ትብሊ/ ትብል?<br>3.3 ኣመራርሓ ልምዓት ጉጅለ ደቂ ኣንስትዮ ሓላፍነተን ንኽዋፅኣ እንታይ ዓይነት ክፍተት ፍልጠት እንተተመለአለን ትብሊ/ ትብል?	ሜላ ምውፅእፃእ ብዕሙቐት ተጠቐም...?
**Thematic 4. ልምዓት ጉጅለ ደቂ ኣንስትዮ ኣብ ክንክን ጥዕና ኣዴታት፣ ሓናጡን ህፃናትን ከይዲ ስልጠና**	
4.1 ናብ ስራሕ መእተዊ ስልጠና ንኣመራርሓ ልምዓት ጉጅለ ደቂ ኣነስትዮ መውሀቢ ከይዲ እንተሃልዩ ምገለፅካለይ/ ምገለፅክለይ ዶ?<br>4.2 እቲ ዝዋሃብ ስልጠና ማለት መጀመሪን ተሃድሶን ከይዲ ከመይ ትዕዘብዮ/ ትዕዘቦ?<br>4.3 ኣብ ሕሉፍ ዓመት ተሃድሶ ስልጠና እንድሕር ዘይወሰዳ፤ ምኽንያት ክትነግርኒ/ ክትነግረኒ ትኽእሊ ዶ?	ብዛዕባ ከይዲ ኣፈፃፅማ ስልጠናን ውፅኢቶምን መሰረት ብምግባር ሜላ ምውፅእፃእ ብዕሙቐት ተጠቐም...?

4.4 ብቲ ዝተውሃበ ወይ ዝዋሃብ ዝነበሩ ስልጠናታት ኣዴታት፣ ሓናጡን ህፃናትን ዝምልከት ንስኺ/ ንስኻ ዕግበትኪ/ ዕጉብ ብኸመይ ትገልፅዮ/ ትገልፆ?	
**Thematic 5. ልምዓት ጉጅለ ደቂ ኣንስትዮ ኣብ ክንክን ጥዕና ኣዴታት፣ ሓናጡን ህፃናትን ከይዲ ሓጋዚ ዑደት**	
5.1 ኣፈፃፅማ ሓጋዛይ ዑደት ሰራሕተኛታት ጥሙር ጥዕና ወይ ሓጋዛይ ዑደት ትገብር ኣካል ኣድላይ ኩነት ኣብ ምምላእን ከይዲን ብኸመይ ትርእዮ/ ትሪኦ?<br>5.2 ብሰራሕተኛታት ጥሙር ጥዕና ዝዋሃበ ሓጋዛይ ዑደት እንታይ ሪኢቶ ኣለኪ/ ኣለካ?	ብዛዕባ ከይዲ ኣፈፃፅማ ሓጋዛይ ዑደትን ውፅኢቶምን መሰረት ብምግባር ሜላ ምውፅእፃእ ብዕሙቐት ተጠቒም...?
**Thematic 6. ልምዓት ጉጅለ ደቂ ኣንስትዮ ኣብ ክንክን ጥዕና ኣዴታት፣ ሓናጡን ህፃናትን ከይዲ ምትብባዕን ወይ ኣፍልጦ ምሃብ**	
6.1 እቲ መተባብዒ ዝዋሃብ ንሓፈሻዊ ኣፈፃፅማ ጥዕና ድዩ? ብመነፅር ስርዓት ጥዕናን ሕብረተሰብን<br>6.2 እዙይ ኣብ ጥዕና ኣዴታት፣ ሓናጡን ህፃናትን ብኸመይ ይፍፀም?<br>6.3 ኣፈፃፅማ መተባቢዒ ወይ ኣፍልጦ ኣዋሃህባ ኣብ ከይዲ ኣገባቡ ፍትሓውነቱን ብኸመይ ትርእዮ/ ትሪኦ?	ኣውፃእፅእ (ብመነፀር: ፍትሓውነቱ፣ መትከላት፣ ኣሳታፋይነት፣ ግልፅነት፣ ተኣማንነትን ካልኦትን)
**Thematic 7. ልምዓት ጉጅለ ደቂ ኣንስትዮ ኣብ ክንክን ጥዕና ኣዴታት፣ ሓናጡን ህፃናትን ንስራሐን ምቹ ዝኾኑ ከባቢን ኣወዳድባን**	
7.4 ኣብ ዲሞክራሲያዊ ኣወዳድባ ልምዓት ጉጅለ እንኮላይ ኣብ ተዋረድ ሓላፍነት ዘለኪ/ ዘለካ ሪኢቶ ምገለፅክለይ/ ምገለፅካለይ ዶ? ብኣውንታN ብኣሉታን ዘለዎ ፅልዋ<br>7.2 ብሰራሕተኛታት ጥሙር ጥዕና ወይ ብኻሊእ ኣካል ኣመራርሓ መርበብ ምትሕግጋዝ ልምዓት ጉጅለ (ሓደ ን ሓሙሽት) ብማዕረ ከም እተን ሓደ ን30 ብማዕረ ኣመራርሓ/ ደገፍ ይረኽባ ዶ እልኪ/ እልካ ትኣምኒ/ ትኣምን?	ብዛዕባ ዲሞክራሲያዊ ውክልናን ውፅኢቶምን መሰረት ብምግባር ሜላ ምውፅእፃእ ብዕሙቐት ተጠቒም...?
**Thematic 8. ኣመራርሓ ልምዓት ጉጅለ ደቂ ኣንስትዮ ኣብ ክንክን ጥዕና ኣዴታት፣ ሓናጡን ህፃናትን ስራሕቲ ኣሚኒታ ኣብ ኩለን ኣባላትን ካልኦት የእትወና ባሃልትን**	
8.1 ኣመራርሓ ልምዓት ጉጅለ ደቂ ኣንስትዮ ኣብ ሕብረተሰብ፣ ኣባላተን፣ ኣብ ባዕለን ኣመራርሓ ልምዓት ጉጅለ ውሺጢ፣ ደቂ ኣነስትዮ ኣመራርሓን ካልኦትን ዘለወን ኣሚኒታ ብኸመይ ትገልፅዮ/ ትገልፆ?	ውፅኢቶም ብኽልቲኡ ገፅ መሰረት ብምግባር ሜላ ምውፅእፃእ ብዕሙቐት ተጠቒም...?
**Thematic 9. ልምዓት ጉጅለ ደቂ ኣንስትዮ ኣብ ክንክን ጥዕና ኣዴታት፣ ሓናጡን ህፃናትን ዘለወን ርክብ ኣብ መንጎኣንን ምስ ካልኦት ዝተፈላለዩ የእትወና ባሃልቲ**	
9.1 ኣፈፃፅማ ስራሕቲ ኣመራርሓ ልምዓት ጉጅለ ንኽመሓየሽ መሳለጥቲ ወይ መዐንቀፍቲ ብብርኪ ሕብረተሰብን መንግስትን ንገለፅክለይ/ ንገለፅካለይ ዶ?<br>9.2 ኣብ ምንታይ ተግባራት ይግለፁ?<br>9.3 ሜላ ርክብ ኣመራርሓ ልምዓት ጉጅለ ምስ የእትወና ባሃልቲ ኣብ **ክንክን ጥዕና ኣዴታት፣ ሓናጡን ህፃናትን** ዝፈጠሮ ብኣውንታ/ ብኣሉታ ምገለፅክለይ/ ምገለፅካለይ ዶ?	ውፅኢቶም ብኽልቲኡ ገፅ መሰረት ብምግባር ሜላ ምውፅእፃእ ብዕሙቐት ተጠቒም...?
ብሓፈሽኡ ኣፈፃፅማ ስራሕቲ ኣመራርሓ ልምዓት ጉጅለ ደቂ ኣንስትዮ ኣብ ሓፈሻዊ ክንክን ጥዕና ብፍላይ ኣብ **ክንክን ጥዕና ኣዴታት፣ ሓናጡን ህፃናትን** ከመይ ትርእዮ?	ሜላ ምውፅእፃእ ብዕሙቐት ተጠቒም...?
ብምጥንኻር ኣፈፃፅማ ልምዓት ጉጅለ፤ ሕብረተሰብ ብቁልጥፍን ብቐፃላይን ውፅኢት ኣብ ክንክን ጥዕና ኣዴታት፣ ሓናጡን ህፃናትን ከረጋግፅ እንታይ ተተገበረ ትብሊ?	ሜላ ምውፅእፃእ ብዕሙቐት ተጠቒም...?

መዘኻኸሪ: ኣብዙይ ክግለፅን ክምመዩን ዝተደለዩ ኣብ ተግባር መጎልበትን መዐንቀፍትን ዝኾኑ ን**ስልጠና፣ ሓጋዚ ዑደት፣ ፍልጠት፣ ኣፍልጦ ምሃብን ንስራሕ ምቹ ዝኾኑ ከባቢን ኣወዳድባን** ክህሉ ኣብ ምግባር ምዝርዛር፡፡ ብተወሳኺ ምንፅፃር ዝተዳለወ ኢደ መጋብርን ብተግባር ዝፍፀም ዘሎን ትፅቢት ትገብረሉ፡፡

መወዳእታ እዙይ መሕትት

ነመስግን፡፡
