# Supplementary material for: Exploring women’s development group leaders’ support to maternal, neonatal and child health care: A qualitative study in Tigray region, Ethiopia
Source: PLoS One. 2021 Sep 23;16(9):e0257602. doi: 10.1371/journal.pone.0257602 (PMC8460027; doi:10.1371/journal.pone.0257602)
Supplement: Attachment — Submitted filename: Response to reviewers_6Aug2021.docx [file pone.0257602.s003.docx]

**Editor’s comments**

**Journal Requirements:**

**Comment:** Please review your reference list to ensure that it is complete and correct. If you have cited papers that have been retracted, please include the rationale for doing so in the manuscript text, or remove these references and replace them with relevant current references. Any changes to the reference list should be mentioned in the rebuttal letter that accompanies your revised manuscript. If you need to cite a retracted article, indicate the article’s retracted status in the References list and also include a citation and full reference for the retraction notice.

**Response: Thank you for this comment. We have reviewed the reference list and ensured that it is complete and correct. We have not used references that have been retracted. The revised manuscript now follows the journal’s referencing requirements.**

When submitting your revision, we need you to address these additional requirements.

**Comment 1**. Please ensure that your manuscript meets PLOS ONE's style requirements, including those for file naming. The PLOS ONE style templates can be found at <https://journals.plos.org/plosone/s/file?id=wjVg/PLOSOne_formatting_sample_main_body.pdf> and https://journals.plos.org/plosone/s/file?id=ba62/PLOSOne_formatting_sample_title_authors_affiliations.pdf

**Response: Thank you for this comment. The manuscript has been revised according to the journal’s style requirements.**

**Comment 2.** When reporting the results of qualitative research, we suggest consulting the COREQ guidelines: <http://intqhc.oxfordjournals.org/content/19/6/349>. In this case, please consider including more information on the number of interviewers, their training and characteristics; and on whether bias issues were considered.

**Response:  We thank you for your guidance. We have added more information on the interviewers and the type of training provided has been added to the manuscript See Methods section, on page 7, line 124. In order to cover diverse perspectives and experiences and reduce the risk of bias, we performed the study in different geographic areas and explored perceptions from different actors, i.e., active women’s development group leaders, health extension workers and managers (See Table 1).**

3. Please provide additional details regarding participant consent. In the ethics statement in the Methods and online submission information, please ensure that you have specified how verbal consent was documented and witnessed.

**Response: We thank you for this guidance. We have provided information on how verbal consent was documented in the manuscript (Methods section, page 9, line 164) and also on the online submission information.**

4. In your Data Availability statement, you have not specified where the minimal data set underlying the results described in your manuscript can be found. PLOS defines a study's minimal data set as the underlying data used to reach the conclusions drawn in the manuscript and any additional data required to replicate the reported study findings in their entirety. All PLOS journals require that the minimal data set be made fully available. For more information about our data policy, please see <http://journals.plos.org/plosone/s/data-availability>.

 Upon re-submitting your revised manuscript, please upload your study’s minimal underlying data set as either Supporting Information files or to a stable, public repository and include the relevant URLs, DOIs, or accession numbers within your revised cover letter. For a list of acceptable repositories, please see <http://journals.plos.org/plosone/s/data-availability#loc-recommended-repositories>. Any potentially identifying patient information must be fully anonymized.

 Important: If there are ethical or legal restrictions to sharing your data publicly, please explain these restrictions in detail. Please see our guidelines for more information on what we consider unacceptable restrictions to publicly sharing data: <http://journals.plos.org/plosone/s/data-availability#loc-unacceptable-data-access-restrictions>. Note that it is not acceptable for the authors to be the sole named individuals responsible for ensuring data access.

 We will update your Data Availability statement to reflect the information you provide in your cover letter.

**Response: Thank you for this comment. We have added the following statement in the Data Availability section:** “**The qualitative data cannot be open access due to confidentiality reasons. De-identified data will be stored on s secure sever at the Ethiopian Publica Health Institute and can be made available upon request by contacting Martha Zeweldemariam** [**martha.zeweldemariam@lshtm.ac.uk**](mailto:martha.zeweldemariam@lshtm.ac.uk)**.” Page 23 line 475.**

 5. Please include captions for your Supporting Information files at the end of your manuscript, and update any in-text citations to match accordingly. Please see our Supporting Information guidelines for more information: http://journals.plos.org/plosone/s/supporting-information.

**Response: We thank you for your guidance. The supporting information captions have been included after the references on page 25.**

 [Note: HTML markup is below. Please do not edit.]

Reviewers' comments:

Reviewer's Responses to Questions

**Comments to the Author**
1. Is the manuscript technically sound, and do the data support the conclusions?

The manuscript must describe a technically sound piece of scientific research with data that supports the conclusions. Experiments must have been conducted rigorously, with appropriate controls, replication, and sample sizes. The conclusions must be drawn appropriately based on the data presented.

Reviewer #1: Yes

2. Has the statistical analysis been performed appropriately and rigorously?

Reviewer #1: N/A

3. Have the authors made all data underlying the findings in their manuscript fully available?

The [PLOS Data policy](http://www.plosone.org/static/policies.action#sharing) requires authors to make all data underlying the findings described in their manuscript fully available without restriction, with rare exception (please refer to the Data Availability Statement in the manuscript PDF file). The data should be provided as part of the manuscript or its supporting information, or deposited to a public repository. For example, in addition to summary statistics, the data points behind means, medians and variance measures should be available. If there are restrictions on publicly sharing data—e.g. participant privacy or use of data from a third party—those must be specified.

Reviewer #1: Yes

4. Is the manuscript presented in an intelligible fashion and written in standard English?

PLOS ONE does not copyedit accepted manuscripts, so the language in submitted articles must be clear, correct, and unambiguous. Any typographical or grammatical errors should be corrected at revision, so please note any specific errors here.

Reviewer #1: Yes

5. Review Comments to the Author

Please use the space provided to explain your answers to the questions above. You may also include additional comments for the author, including concerns about dual publication, research ethics, or publication ethics. (Please upload your review as an attachment if it exceeds 20,000 characters)

Reviewer #1: Thank you for the opportunity to review this manuscript on the WDG in Ethiopia.
I have a few minor suggested edits and a revision which I feel is important for overall understanding of the results.

**Response to the Reviewer:**

Reviewer #1

Line 113 should be “by under-five children” not “of under-five children”

**Response: We thank you for this comment; the change has been made accordingly (page 6, line 106).**

Lines 222-235 the recruitment paragraph in results is confusing and presents conflicting information? It starts by saying community groups are involved in selecting WDG. Then notes Kilte-Awlaelo is an exception but not clear exception to what? The process which looks similar to what was just described? Then the paragraph closes saying communities were not involved in recruitment? Which conflicts with above? This is the one revision which is critical as there is also substantial discussion of this and it is a major conclusion and recommendation. So, the results need to be more clear.

**Response: We thank for these comments. We have adjusted the paragraph so it is clear that women members were not involved in the selection and used the Kilte -Awlaelo as an example for their lack of participation (page 11 line 216).**

Line 269 delete “the” before “women’s”
**Response: We thank you for this comment; the change has been made accordingly (Page 14, line 267).**

Lines 283-284 The sentence result about literate young women would benefit from a quote to support this conclusion.

**Response: We thankful for your valid support, quote has been added to the manuscript (Page 14, line 283).**

6. PLOS authors have the option to publish the peer review history of their article ([what does this mean?](https://journals.plos.org/plosone/s/editorial-and-peer-review-process#loc-peer-review-history)). If published, this will include your full peer review and any attached files.


If you choose “no”, your identity will remain anonymous but your review may still be made public.


**Do you want your identity to be public for this peer review?** For information about this choice, including consent withdrawal, please see our [Privacy Policy](https://www.plos.org/privacy-policy).

Reviewer #1: **Yes:** Debra Jackson

[NOTE: If reviewer comments were submitted as an attachment file, they will be attached to this email and accessible via the submission site. Please log into your account, locate the manuscript record, and check for the action link "View Attachments". If this link does not appear, there are no attachment files.]

While revising your submission, please upload your figure files to the Preflight Analysis and Conversion Engine (PACE) digital diagnostic tool, [https://pacev2.apexcovantage.com/](https://pacev2.apexcovantage.com). PACE helps ensure that figures meet PLOS requirements. To use PACE, you must first register as a user. Registration is free. Then, login and navigate to the UPLOAD tab, where you will find detailed instructions on how to use the tool. If you encounter any issues or have any questions when using PACE, please email PLOS at [figures@plos.org](mailto:figures@plos.org). Please note that Supporting Information files do not need this step.

*In compliance with data protection regulations, you may request that we remove your personal registration details at any time.* [*(Remove my information/details)*](https://www.editorialmanager.com/pone/login.asp?a=r)*. Please contact the publication office if you have any questions.*
